# Bioinspired heliconical auxetic biofibers for intelligent biomechanical surveillance

**DOI:** 10.1126/sciadv.aed6233

**Published:** 2026-06-19

**Authors:** Yi Zhou, Xuechuan Wang, Yifan Wang, Long Xie, Yuanyuan Qiang, Wei Wang, Linbin Li, Ouyang Yue, Xinhua Liu

**Affiliations:** ^1^ College of Chemistry and Chemical Engineering, Shaanxi University of Science and Technology, Xi’an 710021, Shaanxi, China.; ^2^ National Demonstration Center for Experimental Light Chemistry Engineering Education, Shaanxi University of Science and Technology, Xi’an 710021, Shaanxi, China.; ^3^ College of Bioresources Chemical and Materials Engineering, Shaanxi University of Science and Technology, Xi’an 710021, Shaanxi, China.

## Abstract

Wearable bio-based fibers are emerging as platforms for biomechanical sensing and physiological interaction. However, achieving high sensitivity to subtle mechanical cues while maintaining flexibility, self-powered output, and distributed perception remains challenging. Inspired by the helical twining mechanics of climbing plant stems, we design a mechano-adaptive auxetic electronic bio-based fiber as an embodied intelligence sensing unit. The dual-modulus helical confinement, achieved by wrapping rigid aramid fiber/polydimethylsiloxane helices around a flexible collagen aggregate/waterborne polyurethane core, enables programmable deformation and a stable negative Poisson’s ratio (ν = −0.47). Nonlinear intercomponent coupling facilitates synergistic axial-radial dynamics, amplifying interfacial contact variation under strain. The optimized fiber exhibits ultrahigh sensitivity (strain factor: 11.75), strong electrical output (8.1 volts), and remarkable power density (8.48 milliwatts per square meter) with excellent cyclic stability. Integrated into fiber-sensing arrays, it decodes microstrain patterns linked to lower-limb function, offering a scalable strategy for self-powered, adaptive, and durable biomechanical monitoring.

## Teaser

Bioinspired auxetic fibers enable self-powered, intelligent surveillance of subtle biomechanical activities.

## INTRODUCTION

Wearable electronic fibers have emerged as a promising foundation for distributed body-sensing networks, serving as the physical substrate for embodied intelligence that enables perception-action integration across the human body, enabling continuous acquisition of biomechanical information across multiple body regions (*1*–*3*). Their inherent softness, flexibility, and compatibility with textile architectures make them particularly attractive for constructing large-scale, conformal sensing systems capable of tracking complex physiological motions in real time (*4*, *5*). However, achieving high sensitivity to subtle, localized deformations while maintaining mechanical adaptability and self-powered operation remains an unresolved challenge (*6*–*8*). The weak coupling between mechanical and electrical domains in most soft materials limits their responsiveness to small-amplitude strains, constraining their application in distributed biomechanical sensing and long-term wearable monitoring (*9*–*11*). For instance, early manifestations of venous dysfunction, such as minor wall strain fluctuations or valve motions, offer a compelling example of the need for such precision detection (*12*–*14*). The failure of existing electronic fibers to capture these subtle biomechanical cues is primarily because of their insufficient strain sensitivity and limited interfacial dynamics during deformation (*15*, *16*). Consequently, a strategy that simultaneously enhances microstrain responsiveness and maintains structural adaptability is urgently needed for next-generation wearable sensing systems (*17*–*19*).

Nature provides profound inspiration for solving this materials paradox. Twining stem plants exhibit directional helical winding architectures that distribute mechanical stress uniformly while preserving flexibility and strong adhesion to irregular surfaces (*20*, *21*). These bioinspired helicoidal geometries, characterized by dynamic anisotropy and high surface area, enable coordinated axial-radial deformation—a mechanism that efficiently converts mechanical perturbations into functional responses (*22*, *23*). Translating this principle into synthetic fibers offers a pathway to integrate mechanical adaptability and efficient energy transduction within a single, continuous structure (*24*, *25*). Auxetic structures, which exhibit a negative Poisson’s ratio, provide a powerful design framework to amplify deformation effects by expanding laterally under stretch, which enhances interfacial contact for sensing (*26*–*30*). Although high-sensitivity microstrain sensing has been achieved in non-auxetic fibers via nanofiber buckling mechanisms by Lin *et al.* (*31*), the exploitation of auxetic architectures for detecting subtle physiological signals remains a distinct gap. Most existing auxetic designs, such as the seminal textile triboelectric nanogenerators (TENG) by Chen *et al.* (*32*), are predominantly optimized for macroscopic energy harvesting rather than the high sensitivity required for microstrain detection. This highlights a prevalent focus in the field: Most existing auxetic designs are based on rigid lattices optimized for macroscopic stiffness and large deformations, rather than the high sensitivity required in microstrain sensing (*33*, *34*).

Here, we introduce a mechano-adaptive auxetic electronic bio-based fiber that synergistically integrates bioinspired helical topology with triboelectric transduction as an embodied intelligence sensing unit. Via a dual-modulus helical confinement strategy, rigid aramid fiber/polydimethylsiloxane (AF/PDMS) fibers are wrapped around a flexible nature collagen aggregate/waterborne polyurethane elastomer (CA/WPU) core fiber, creating a hierarchical architecture with a stable negative Poisson’s ratio. This strategic material hierarchy enables coordinated axial-radial deformation, amplifying interfacial contact and charge transfer under strain. The fiber achieves high strain sensitivity (factor = 11.75) and power density (8.48 mW m^−2^). Integrating it into sensing textiles enables the use of machine learning for biomechanical mapping and adaptive signal interpretation, establishing an intelligent connection between microstrain patterns and physiological functions. This provides a universal strategy for self-powered and long-term monitoring of electronic fibers.

## RESULTS

### The structure and design principle of auxetic fibers

**Fig. 1.**
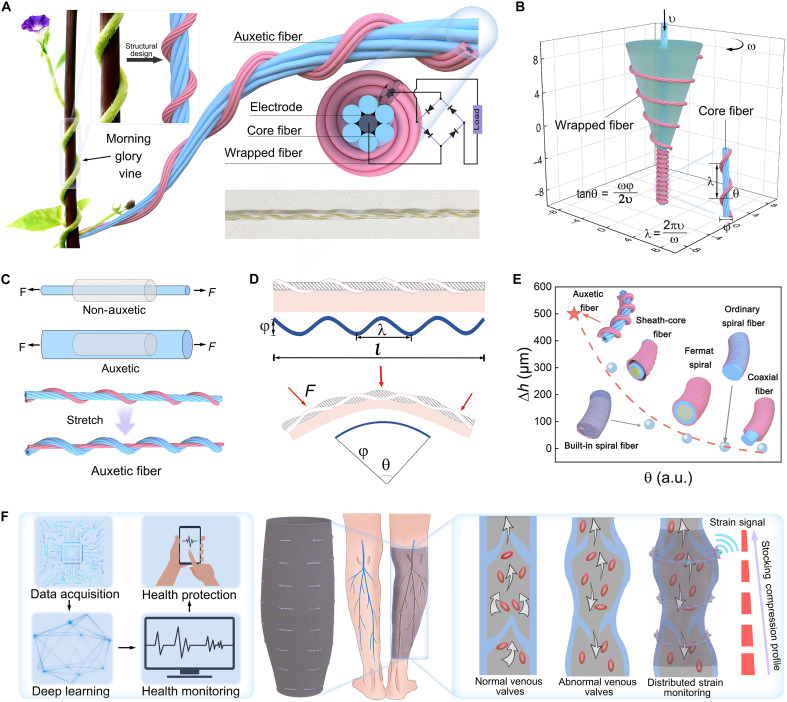
Schematic illustration of the working mechanism and structural design of the auxetic sensing fiber. (**A**) Bioinspired design principle of the auxetic fiber, inspired by the high–contact area structure of a morning glory vine. (**B**) Twist-based manufacturing strategy for auxetic fiber. (**C**) Comparison of deformation behavior under axial strain: conventional (non-auxetic) fiber exhibiting radial contraction versus auxetic fiber showing radial expansion. (**D**) Skin conformability and interfiber spacing variation of auxetic fiber during deformation. (**E**) Comparative analysis of interfiber triboelectric gaps among different fiber architectures: coaxial, ordinary spiral, built-in spiral, sheath-core, and auxetic fibers. a.u., arbitrary units. (**F**) Left: Functional schematic of a smart compression stocking knitted with auxetic fibers for health monitoring; Right: Illustration of venous valve states (normal versus abnormal) and the integrated sensing mechanism compatible with the stocking’s gradient compression profile.

Inspired by the climbing mechanism of twining plants such as the morning glory vine, which achieves stable attachment through helically coiled stems, we designed a bioinspired mechano-adaptive auxetic electronic fiber by wrapping a yarn around a core filament at a defined helical angle (Fig. 1A). This structural strategy imitates the plant’s efficient use of geometry to maximize surface contact, resulting in enhanced and uniform interfacial adhesion. To realize continuous fabrication, a custom elastic winding system was developed (Fig. 1B). A large-diameter, high-elasticity CA/WPU filament served as the core, while a smaller-diameter, high-tensile AF/PDMS fiber acted as the wrapping component. The core fiber was drawn axially via bottom traction, while the wrap fiber was dispensed from a rotating bobbin and helically wound onto the core. The precise helical geometry is governed by the kinematic relationship between the core feed rate (υ) and the bobbin’s angular velocity (ω) (note S4). According to the helical path kinematics, the resulting helical pitch (λ) and winding angle (θ) are determined by

$$\lambda =\frac{\pi \varphi }{\text{tan}\theta }$$ (1)

where λ is the helical pitch of auxetic fiber, θ is the winding angle between the wrapped fiber and the horizontal axis of the core fiber, φ is the diameter of the resulting auxetic fiber, ω is the angular velocity of the wrapped fiber, and υ is the feed rate of the core fiber.

The auxetic behavior arises from the material’s distinctive internal architecture (Fig. 1C). Unlike conventional structures that contract laterally when stretched, the auxetic configuration expands transversely under tension, providing enhanced resistance to deformation. This negative Poisson’s ratio behavior increases the interfiber contact distance during stretching, ensuring stable and extensive contact between triboelectric layers (Fig. 1D). As tensile strain rises, the helical configuration induces directional interfacial sliding, which strengthens charge transfer efficiency. This dynamic mechanism amplifies subtle muscle microstrains into measurable displacements, thereby improving the conversion of mechanical input into electrical output (*35*). The relationship between fiber deformation and triboelectric voltage is expressed by the following equation, with details provided in note S5

$$A_{1}=2n\sqrt{r_{w}^{2}-{\left(d_{0}-\beta \varepsilon -r_{c}\right)}^{2}}\frac{\lambda \left(1+\varepsilon \right)}{\text{cos}\theta }$$ (2)

At higher strain levels, the wrapped and core fibers transition into a dual-helix configuration, where the wrapped fiber begins to transpose relative to the core under tensile loading. The contact area in this stage is governed by the geometry of the intertwined helices, taking interfacial deformation into account. The evolution of this area follows the relationship described by

$$A_{2}=nk_{w}{\left[2\sqrt{r_{w}+r_{c}+\delta^{2}-{\left(d_{0}-\beta \varepsilon \right)}^{2}}\right]}^{2}\frac{\lambda \left(1+\varepsilon \right)}{\text{cos}\theta }$$ (3)

where n is the number of wrapped fibers, $r_{w}$ and $r_{c}$ are the radii of the wrapped and core fibers, respectively, $d_{0}$ is the initial center-to-center distance between the wrapped and core fibers, β is the reduction in center distance during straining, ε is the applied axial strain, λ is the initial helical pitch, θ is the helical angle, $k_{w}$ is a dimensionless scaling factor for the contact width in the second stage, and δ is the initial contact deformation.

Upon axial stretching, the high-modulus wrapped fiber tends to straighten, reducing its helical angle and exerting radial compression on the softer core. This deformation enlarges the real contact area between the two fibers, altering the distribution and density of microcontact points. Variations in these contact points modulate the triboelectric potential and generate current in the external circuit. As stretching continues, the wrapped fiber undergoes slight axial sliding, creating new contact interfaces and refreshing existing ones. The combination of variable contact spacing and controlled friction substantially enhances output performance (fig. S1). The effective contact distance during deformation can be expressed as, with details provided in note S6

$$d=\left[1-\frac{A}{2\pi r_{w}L\left(1+\varepsilon \right)}\right]\left(d_{0}-\beta \varepsilon \right)-\left(r_{c}+vd_{0}-r_{w}\right)$$ (4)

where L is the effective length of the fiber, v is the Poisson’s ratio of the fiber structure, $r_{w}$ and $r_{c}$ are the radii of the wrapped and core fibers, respectively, $d_{0}$ is the initial center-to-center distance between the fibers, β is the reduction in the center distance during straining, and ε is the applied axial strain.

Analysis of the frictional gap and strain energy distribution across different fiber configurations (Fig. 1E and fig. S2) demonstrates that the proposed auxetic design achieves the largest interfiber frictional gap reported in fiber-based sensors (*31*, *36*–*39*). This architecture effectively mitigates the typical trade-off between stretchability and electrical output, leading to stable, high-performance sensing under dynamic deformation. Building on this structural innovation, we implemented an auxetic yarn–based sensing system for early detection of venous dysfunction (Fig. 1F). Venous stasis often induces subtle microstrains in leg muscles, which are difficult to detect with conventional sensors. To address this, we designed a transverse array of eight auxetic yarns with graded mechanical sensitivity, integrated into a compression textile. The modulus of the auxetic yarn is engineered to increase progressively from the distal to the proximal region (fig. S3). This gradient mirrors the stiffness profile of compression stockings to ensure consistent sensitivity across distinct pressure zones. By manipulating the helical angle and pitch of the wrapping yarn, we achieved a synergistic integration. This design maintains mechanical compatibility with the stocking’s compression gradient and simultaneously enables the real-time detection of venous microdeformations. This adaptive sensing strategy marks a transition from reactive diagnosis to proactive physiological monitoring. It thereby establishes a digital framework for early intervention in venous disorders.

### Fabrication and mechano-electric performance of the auxetic fiber

**Fig. 2.**
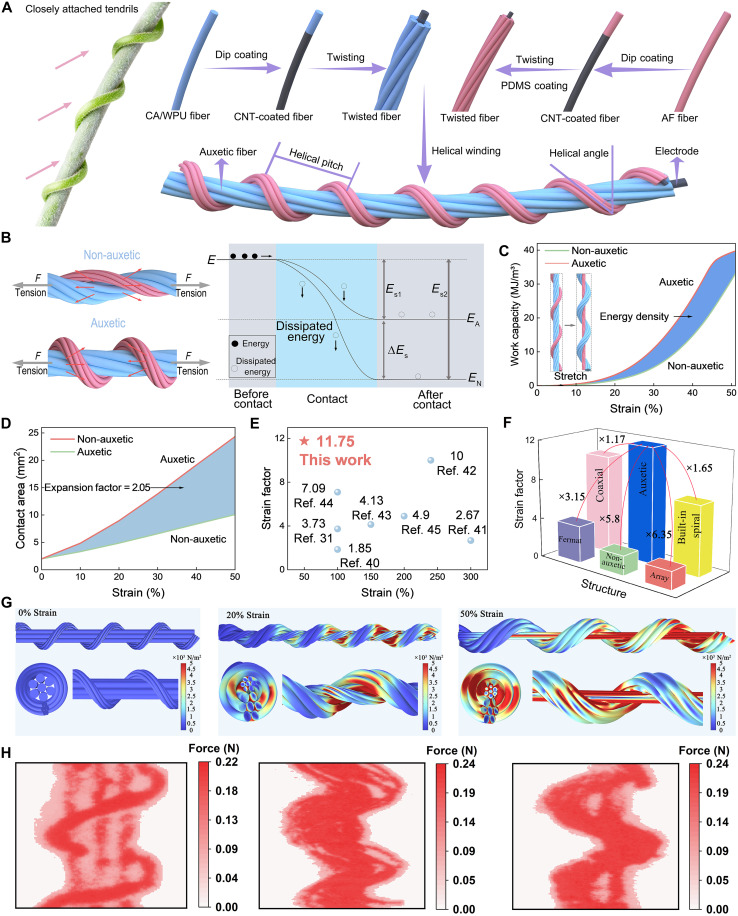
Performance comparison of non-auxetic and auxetic fiber–based metamaterials. (**A**) Schematic diagram illustrating the fabrication process of the auxetic fiber. (**B**) Left: Tensile resistance behavior of non-auxetic and auxetic fiber metamaterials under radial stretching, indicating the direction of internal force dissipation. Right: Schematic of energy dissipation in non-auxetic versus auxetic metamaterials. (**C**) Work capacity of non-auxetic and auxetic fiber metamaterials at 50% strain, tested using fibers of equal length with helical pitches of 5 mm (auxetic) and 2 mm (non-auxetic). (**D**) Variation in contact area between non-auxetic and auxetic fiber metamaterials within 50% strain. (**E**) Comparison of the strain coefficient between the present study and state-of-the-art reported findings, normalized to the signal amplitude variation rate under identical stimulus conditions. (**F**) Relationship of the strain coefficient among auxetic fiber structure and other triboelectric fiber architectures: coaxial, Fermat spiral, built-in spiral, array, and non-auxetic. (**G**) COMSOL simulation of the stress distribution in the auxetic fiber metamaterial under uniaxial radial stretching, showing the overall stress, axial stress, and enlarged view of a single pitch. (**H**) Pressure imaging maps corresponding to three key stages of fiber deformation under stretching.

The fabrication process of the auxetic fiber is illustrated in Fig. 2A. The fiber features a helically wrapped auxetic structure inspired by the directed tight winding mechanism of twining plants, composed of a CA/WPU composite core and an AF/PDMS sheath. Multiple strands of the AF/PDMS yarn were helically wrapped around the CA/WPU core at a predefined pitch and angle, forming a stable auxetic architecture. This twist-assisted process was optimized for uniformity and scalability, as verified by gravimetric analysis and microscopic imaging (figs. S7 to S9).

Under axial tension, the auxetic fiber displays a distinct deformation mechanism compared with conventional (non-auxetic) structures (Fig. 2B). This behavior is fundamentally driven by our dual-modulus helical constraint strategy, where a high-modulus wrap tightly constrains a low-modulus core. The internal stress is redistributed inward along the helical axis, reducing energy dissipation and increasing local contact forces between the triboelectric layers. This configuration enables more efficient conversion of mechanical strain energy into electrical output. As shown in Fig. 2C, the auxetic fiber exhibits a substantially higher work capacity at identical strain levels, confirming improved electromechanical coupling efficiency. The enhanced performance originates from the geometry-induced expansion behavior characteristic of auxetic materials. When stretched, the structure undergoes lateral expansion, increasing the effective contact area between the core and wrapped layers. Quantitative analysis reveals a 2.05-fold increase in triboelectric contact area compared to the non-auxetic structure (Fig. 2D), providing favorable conditions for charge generation and energy harvesting. To further evaluate strain responsiveness, we compared the normalized strain factor and fabrication efficiency of our auxetic fiber with reported stretchable fiber sensors (Fig. 2E and tables S1 and S2). The auxetic fiber achieves a strain factor of 11.75, surpassing most previously reported designs (*40*–*45*). This outstanding sensitivity arises from its helical wrapping geometry and stress-focusing topology, which amplify microscale strain variations into detectable electrical signals. Unlike traditional fiber sensors that depend on nanostructured surfaces or interfacial separation, this design achieves efficient stress-signal transduction under small deformations, effectively overcoming the trade-off between flexibility and sensitivity (Fig. 2F).

To quantify this enhancement, we modeled the interfacial contact pressure (*P*) as a function of the Poisson’s ratio difference (note S7). Theoretically, the pressure is governed by the modulus mismatch, satisfying

$$P\approx E_{\text{core}}\varepsilon_{Z}\left(\nu_{\text{core}}-\nu_{\text{struct}}\right)$$ (5)

where *E*_core_ is the Young’s modulus of the core fiber, $\varepsilon_{Z}$ represents the applied axial tensile strain, $\nu_{\text{core}}$ is the Poisson’s ratio of the core fiber, and $\nu_{\text{struct}}$ denotes the equivalent structural Poisson’s ratio of the helical wrap. This derivation indicates that a substantial modulus difference is the prerequisite for generating sufficient interfacial contact pressure (fig. S10).

To validate this criterion, we performed COMSOL finite element analyses comparing structures with different modulus configurations. As shown in fig. S11, low-modulus ratio structures exhibit a flat and negligible pressure distribution. In sharp contrast, the high-modulus mismatch design induces a strong stress concentration at the helical interface (Fig. 2G). Crucially, this localized stress translates directly into enhanced electrical output, as substantiated by the triboelectric charge transfer modeling (fig. S12). Simulations reveal that surface charge density peaks precisely at these high-stress contact zones, confirming the stress-driven electromechanical coupling mechanism. In addition, pressure mapping during three characteristic deformation stages (Fig. 2H) reveals dynamic rearrangement between the core and sheath fibers, illustrating the continuous evolution of the auxetic architecture under strain.

### Mechanism of triboelectric response in auxetic fibers

**Fig. 3.**
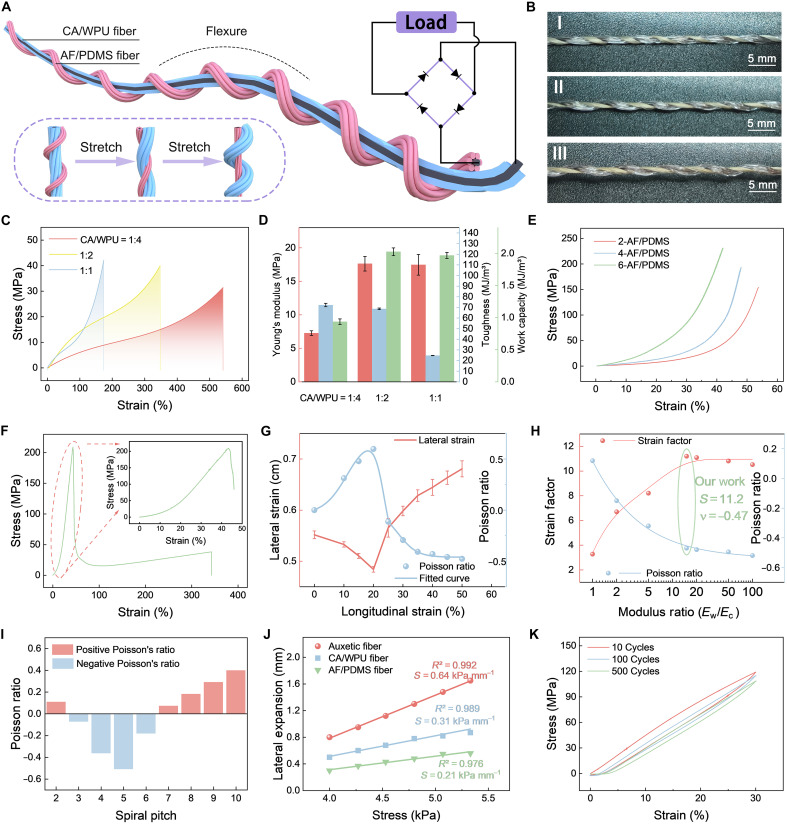
Structural and mechanical performance optimization of auxetic fiber. (**A**) Schematic of auxetic fiber structure; inset illustrates the distinct stages of fiber stretching. (**B**) Optical images capturing the fiber morphology at different tensile stages. (**C**) Stress-strain curves of CA/WPU core fibers with different blending ratios. (**D**) Young’s modulus, toughness, and work capacity of CA/WPU core fibers as a function of composition. (**E**) Stress-strain behavior of AF/PDMS wrapped fibers. (**F**) Representative tensile curve of auxetic fiber, showing characteristic two-stage failure. (**G**) Evolution of axial strain and Poisson’s ratio during radial expansion of auxetic fiber. (**H**) Effect of modulus ratio on the strain factor and Poisson’s ratio. (**I**) Poisson’s ratio of the auxetic fiber under varying helical pitches. (**J**) Transverse expansion of auxetic fiber under varying pressure levels. (**K**) Flexural fatigue testing of auxetic fiber.

Benefiting from its unique macroscopic negative Poisson’s ratio structure, auxetic fiber exhibits pronounced radial expansion upon axial stretching. As illustrated in Fig. 3A, the triboelectric response mechanism uses a CA/WPU fiber as the core (positive triboelectric material) and an AF/PDMS fiber wrapped around it as the sheath (negative triboelectric material), and the conductive yarn coated with carbon nanotubes is used for conductivity. When the fiber is stretched axially, the high-modulus sheath fiber tends to straighten due to its high resistance to elongation, exerting circumferential compressive stress on the low-modulus core. This induces helical buckling deformation in the core, while the reduction in the helical angle of the wrapped filaments converts axial strain energy into radial expansion via geometric coupling, resulting in a pronounced auxetic effect. This deformation mechanism promotes relative displacement and interfacial exchange between the core and sheath fibers, markedly enhancing interfiber charge transfer efficiency. Optical images of the fiber under progressive stretching are presented in Fig. 3B.

To optimize mechanical performance, we varied the CA/WPU composition of the core. Increasing WPU content raised breaking strain while lowering tensile strength (Fig. 3C). Correspondingly, higher collagen fraction reduced Young’s modulus but increased work capacity—defined as energy absorbed per unit volume—consistent with enhanced energy dissipation via collagen-polyurethane hydrogen bonding (Fig. 3D). The sheath fiber must have high tensile strength to effectively constrain the core fiber during deformation. As shown in Fig. 3E, increasing the number of sheath fiber strands notably enhanced the tensile performance: The external PDMS layer primarily accommodated deformation in the initial stage, while the internal AFs progressively bore the load with increasing strain, achieving a maximum tensile strength of 240 MPa. A typical tensile curve of the auxetic fiber exhibited a two-stage fracture characteristic (Fig. 3F), initial rupture of the high-strength, low-elongation sheath fiber followed by fracture of the highly extensible core fiber, showing a tensile strength of 207.6 MPa and an elongation at break of 340%. Radial diameter measurements (Fig. 3G) reveal typical Poisson contraction at strains below 20%. Beyond this threshold, further elongation induces structural reconfiguration, leading to substantial radial expansion and a pronounced auxetic effect. This transverse expansion stems primarily from the mechanical mismatch between the yarn components. As shown in fig. S13, the sheath exhibits a considerably higher Young’s modulus than the soft core. This stiffness disparity forces the sheath to laterally compress the core during elongation, driving overall structural expansion. Critically, the strain factor and Poisson’s ratio of the fiber depend heavily on the modulus ratio (Fig. 3H and table S4). Increasing the modulus contrast effectively regulates the structural evolution, promoting the transition from non-auxetic to auxetic behavior. This mechanism optimizes the contact area and contact pressure during stretching, thereby greatly enhances the sensitivity and electrical output of the fiber.

Besides the material properties, the geometric structure also plays a vital role. The helical pitch of the wrapped structure is a key parameter governing the auxetic behavior. Optical images of fibers with different helical pitches are shown in figs. S14 and S15. As illustrated in Fig. 3I and fig. S16, the fiber exhibited auxetic behavior within a helical pitch range of 3 to 6 mm, with optimal mechanical performance observed at 4 mm. However, maximizing macroscopic deformation does not strictly equate to maximizing the final electrical output for a functional sensing fiber. The final device incorporates an adjusted pitch of ~5.0 mm to achieve the best comprehensive performance. This adjustment optimally balances structural deformation with triboelectric charge generation as detailed in the subsequent electrical analysis. Furthermore, the fiber exhibited high compressive sensitivity (0.64 kPa mm^−1^; Fig. 3J), indicating its potential for multimode mechanical sensing. Auxetic fiber also demonstrated excellent cyclic tensile stability, maintaining consistent performance after 500 stretching cycles at 30% strain (Fig. 3K). Through rational structural design and compositional tuning, we have successfully fabricated a smart fiber that combines a robust auxetic effect, high linear response, and superior triboelectric output, providing an important material foundation for textile-based strain sensing fibers.

### Performance of auxetic fiber in microstrain sensing

**Fig. 4.**
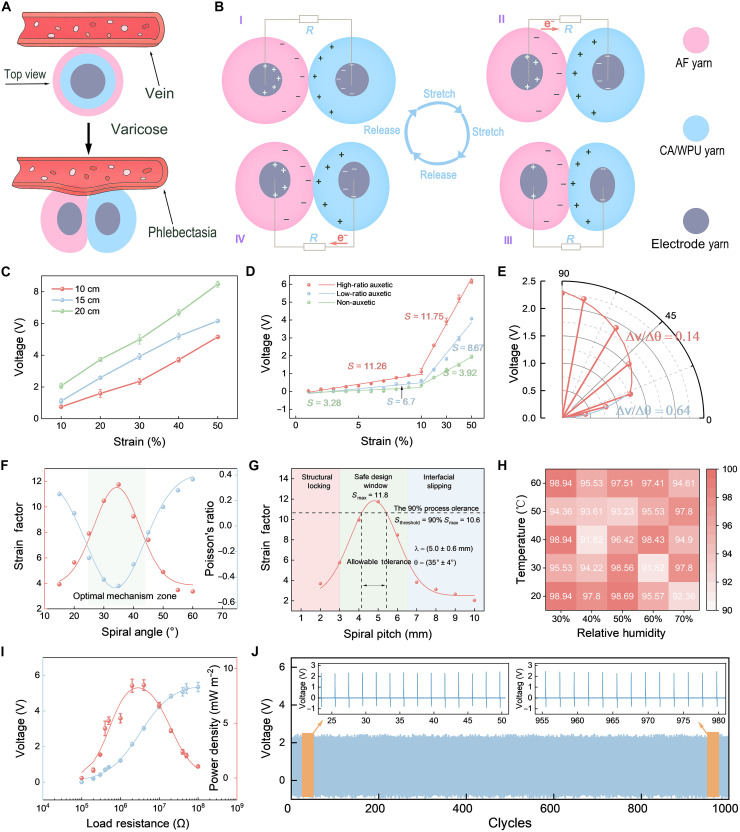
Response mechanism and performance optimization of auxetic fiber. (**A**) Schematic illustrating auxetic fiber’s deformation mechanism under venous dilation. (**B**) Mechanism of triboelectric charge transfer in auxetic fiber under microscale strain. (**C**) Output voltage of auxetic fiber under different lengths during stretching. (**D**) Quantitative analysis of strain factor across the full deformation range (0 to 50%). (**E**) Voltage output of auxetic fiber under different bending angles. (**F**) Effect of the helical angle (θ) on the strain factor and structural Poisson’s ratio. (**G**) Impact of helical pitch on strain factor and fabrication tolerance. (**H**) Environmental stability of auxetic fiber’s output under varying temperature and humidity conditions. (**I**) Voltage and power density of auxetic fiber as a function of external load resistance. (**J**) Cyclic stability of the fiber under repeated stretching (strain: 20%; cycles: 1000).

Benefiting from its distinctive tensile response, the auxetic fiber provides an effective platform for detecting subtle mechanical deformations in the human body. As shown schematically in Fig. 4A, the fiber forms a conformal interface with muscle tissue and responds in real time to the microscopic strains induced by venous obstruction. Its auxetic topology efficiently transmits local strain variations, translating minor biomechanical deformations into measurable electrical outputs. The charge transfer process depends on the degree of strain (Fig. 4B): At low deformation, interfacial electrification occurs mainly through contact sliding, whereas at higher strain, yielding of the core fiber leads to positional exchange between the inner and outer filaments, amplifying charge generation (fig. S2). This strain-driven expansion of the contact area mitigates charge dilution—commonly observed in conventional stretchable triboelectric systems—and markedly enhances charge transfer efficiency. Finite-element simulations confirmed the strain-induced charge redistribution and local electric field enhancement within the auxetic structure (fig. S17).

The electrical output increased proportionally with fiber length (Fig. 4C, movie S1, and fig. S18), reaching a peak open-circuit voltage of 8.1 V for a 20-cm sample. To quantitatively evaluate structural advantages across the entire deformation range, we analyzed the voltage-strain curves of the high-modulus ratio auxetic fiber, the low-modulus ratio auxetic fiber, and the non-auxetic twisted fiber. As shown in Fig. 4D and fig. S19, in the large strain regime (10 to 50%), the high-modulus ratio design maintained a high strain coefficient of 11.75. This performance clearly outperforms both the low-modulus ratio auxetic fiber and the non-auxetic control, validating the effectiveness of the stress concentration induced by modulus mismatch over a wide tensile range. Crucially, this structural advantage is further amplified in the low-strain regime (0 to 10%), where the high-modulus ratio auxetic fiber achieves a remarkable strain coefficient of 11.26. This value is approximately 3.4 times that of the non-auxetic fiber, indicating a broader effective response range. These results not only reflect the device’s superior responsiveness to minute deformations but also compellingly demonstrate the pivotal role of an optimized modulus ratio in maximizing electromechanical conversion efficiency. It also demonstrated excellent bending performance (Fig. 4E), producing stable signals over angles from 5° to 90° with a bending sensitivity coefficient of 0.64, which compares favorably with previously reported one-dimensional sensing fibers (fig. S20 and table S3) (*31*, *46*–*48*).

To establish design rules for maximizing sensing performance, we systematically optimized the helical geometry using the manufacturing parameter relationships derived in Eq. 1. Figure 4F and fig. S21A illustrate the correlation between the helical angle, output sensitivity, and the structure’s Poisson’s ratio. Notably, the strain factor peaks within the optimal mechanism zone (25° < θ < 40°). This region coincides with the minimum structural Poisson’s ratio, confirming that the maximized auxetic effect is the physical origin of the high sensitivity. Deviating from this optimal range causes a rapid decay in performance. Specifically, an excessively small angle results in insufficient structural opening, while an excessively large angle diminishes the effective radial contraction efficiency. Beyond the helical angle, fig. S21B quantifies the influence of helical pitch on the electrical performance. The highest output voltage is achieved at a moderate pitch of 5.0 mm. This result differs from the mechanically optimal pitch of 4 mm identified in Fig. 3I. This deviation arises because the electrical output relies heavily on the effective interfacial contact area generated dynamically during stretching, rather than depending solely on macroscopic deformation. The 5.0-mm pitch provides an optimal balance between the structural contraction of the auxetic design and the interfacial contact. Consequently, constraining the pitch to this electrically optimal target of 5.0 mm is essential for maximizing electrical signal generation. In parallel with geometric optimization, dimensional compatibility was also critical. As shown in fig. S22, a wrapped-to-core diameter ratio of 1:2 yielded the highest triboelectric output voltage, demonstrating that this specific ratio optimally balances efficient charge generation with auxetic deformation. Integrating these geometric and dimensional constraints, Fig. 4G maps the strain factor against manufacturing tolerances to verify stability. Incorporating these parameters allowed us to define a safe design window (λ = 5.0 ± 0.6 mm, θ = 35 ± 4°). Operating within this window ensures an optimal auxetic effect and mitigates the impact of minor geometric deviations, providing robust guidelines for scalable manufacturing.

Beyond sensitivity, dynamic response capability is essential for capturing real-time human motion. Figure S23 displays the sensor output across a frequency range of 0.5 to 2.5 Hz, which simulates typical human step frequencies. Within this bandwidth, the device exhibited a highly linear response (*R*^2^ = 0.98). Moreover, waveform analysis reveals a fast response time of 50 ms and a recovery time of 70 ms. These temporal characteristics satisfy the requirements for detecting rapid physiological signals and gait phases. The fiber maintained excellent environmental stability across varying temperature and humidity levels, achieving a correlation coefficient above 92% in the response matrix (Fig. 4H and fig. S24). Load-dependent measurements indicated a maximum power density of 8.48 mW m^−2^ at an external resistance of 4 megohm, while long-term cycling confirmed outstanding durability—the electrical signal remained consistent after 1000 stretching cycles at 20% strain (Fig. 4, I and J). Through the synergistic combination of mechanical adaptability, structural optimization, and robust triboelectric output, the auxetic fiber demonstrates exceptional sensitivity, environmental resilience, and long-term reliability. These attributes establish it as a promising candidate for continuous microstrain monitoring and energy-adaptive wearable sensing systems.

### Application of auxetic fiber in lower-limb venous status monitoring

**Fig. 5.**
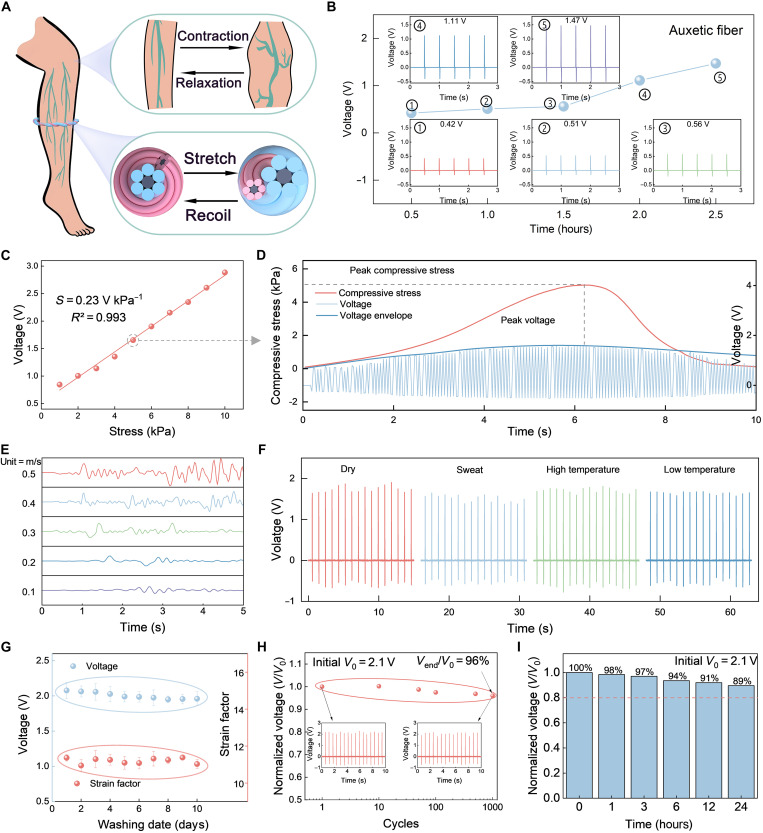
Applied monitoring performance of auxetic fiber. (**A**) Working principle of auxetic fiber for monitoring muscle contraction and relaxation induced by venous dysfunction. (**B**) Output performance of auxetic fiber during prolonged sitting (2.5 hours), showing signal accumulation due to venous pressure variation. (**C**) Pressure sensitivity of auxetic fiber under external loading conditions. (**D**) Cyclic loading-unloading curves at 5 kPa, showing the synchronous voltage output and envelope trend. (**E**) Output signal response of auxetic fiber under simulated blood flow at different velocities. (**F**) Environmental stability of auxetic fiber under varying temperature and humidity conditions. (**G**) Washing durability tested over 10 days (3 cycles per day, 20 min per cycle). (**H**) Chemical stability evaluation under continuous immersion in artificial sweat for 24 hours. (**I**) Mechanical abrasion resistance test over 1000 cycles.

The high microstrain sensitivity of the auxetic fiber enables precise monitoring of biomechanical signatures associated with venous status in the lower limbs. Its responsiveness in the low-strain regime allows effective coupling with physiological activities such as muscle contraction-relaxation and localized tissue volume fluctuations, capturing subtle mechanical cues associated with lower-limb volumetric strain (Fig. 5A). As shown in Fig. 5B, the fiber was attached to the lower leg for continuous monitoring over 2.5 hours in a seated posture. The output voltage gradually increased with gravity-induced volumetric expansion, clearly reflecting the mechanical strain variations of lower limb tissues under static conditions and confirming the system’s reliability during real-time wear. The fiber also exhibited stable pressure sensing with a sensitivity of 0.23 V kPa^−1^ (Fig. 5C and fig. S25). Under cyclic loading and unloading at 5 kPa, the output signals closely followed the input waveform (Fig. 5D), indicating excellent repeatability and synchronization. We further verified the signal stability under varying tensile states (fig. S26). The auxetic fiber retained >96% of its sensitivity within the physiological range (0 to 20% strain) and >86% even under extreme deformation (50%). This confirms that macroscopic tissue stretching does not compromise the detection of subtle microstrains. To further assess performance under simulated physiological flow, a bioinspired fluid pressure testing platform was developed (fig. S27 and movie S2). The output voltage increased monotonically with fluid velocity (0.1 to 0.5 m s^−1^; Fig. 5E) and exhibited high linearity. This linear response facilitates the quantitative calibration of flow-induced mechanical strains. It further validates the efficient electromechanical coupling of the fiber and its suitability for capturing dynamic mechanical signatures in the microstrain regime (*49*, *50*).

To assess reliability under practical wearable conditions, we evaluated the sensing stability across diverse environments. As summarized in Fig. 5F, voltage variation remained below 5% across a temperature range of 10° to 40°C and humidity levels of 30 to 80%, demonstrating robust environmental adaptability. We further subjected the fiber to rigorous durability tests. After multiple washing cycles (Fig. 5G), the device retained its voltage output and sensitivity with a performance degradation of less than 6%. This stability highlights its durability for long-term wearable applications. Furthermore, the device exhibited exceptional mechanical robustness, maintaining 96% of its initial performance after 1000 abrasion cycles (Fig. 5H). Such resilience ensures stable operation against the surface friction encountered during daily wear. The fiber also demonstrated superior chemical stability when immersed in artificial sweat, retaining ~89% of its signal output after 24 hours of continuous exposure. This resistance to ionic corrosion is attributed to the effective encapsulation provided by the hydrophobic PDMS sheath (Fig. 5I). Last, the system clearly differentiated between resting and active states (fig. S28). Collectively, these results establish the auxetic fiber as a highly sensitive, stable, and durable platform for the real-time assessment of lower limb function.

**Fig. 6.**
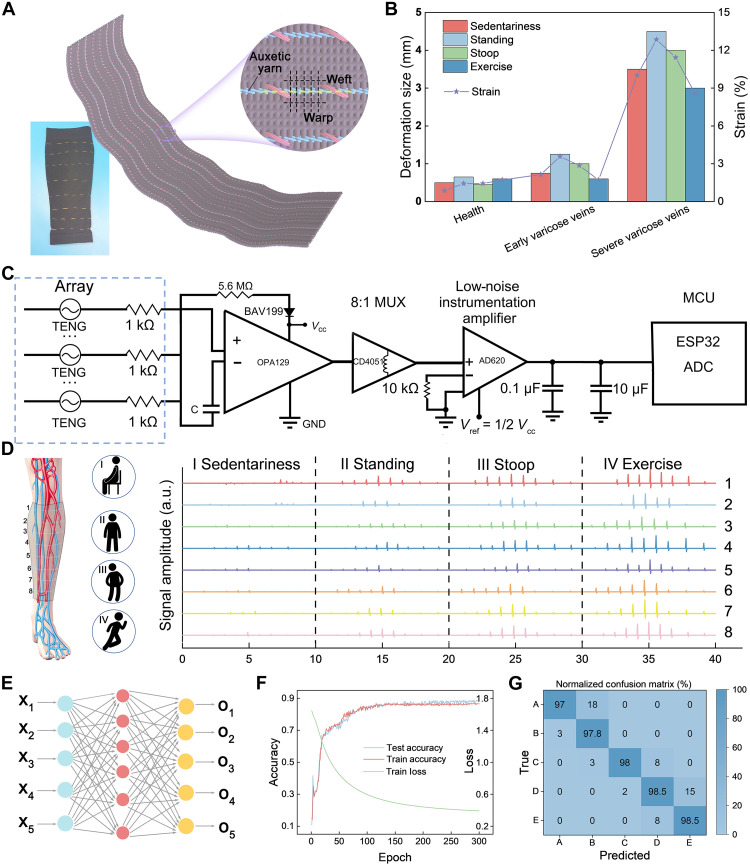
System integration and intelligent data analysis for venous function assessment. (**A**) Photograph of the final smart stocking prototype integrated with the sensing fiber array, with an inset showing the detailed knitting structure. (**B**) Quantified transverse deformation and strain distribution of the lower limb under different physiological states. (**C**) System-level block diagram of the multichannel data acquisition system. (**D**) Representative multichannel signal matrix recorded during lower-limb activities: prolonged sitting (I), prolonged standing (II), prolonged stooping (III), and exercise (IV). (**E**) Architecture of the five-layer artificial neural network (ANN) model for physiological state classification from complex signal matrices. (**F**) Training process of the ANN model, showing the evolution of accuracy and loss function over epochs. (**G**) Normalized confusion matrix summarizing the classification performance of the trained ANN model. GND, ground; MUX, multiplexer; MCU, microcontroller unit; ADC, analog-to-digital converter.

The fiber’s flexibility and stretchability allow seamless integration into textile structures. As illustrated in Fig. 6A, it was knitted into a commercial compression stocking, forming an auxetic textile capable of accommodating complex deformations. Conventional fibers typically exhibit radial contraction and localized pressure accumulation under tension. Conversely, the auxetic fiber expands transversely. This mechanism ensures uniform pressure distribution and high dynamic compliance (fig. S29). Consequently, the textile maintains intimate skin contact without restricting natural muscle expansion. This configuration is particularly advantageous for detecting the subtle strain variations associated with biomechanical variations relevant to venous health. Figure 6B presents the characteristic transverse strain distribution of the lower limb under healthy, mild varicose, and severe varicose conditions, underscoring the need for precise sensing in the microstrain regime (*51*–*53*). By embedding the auxetic fibers into the compression stocking, effective detection of localized tissue strain was achieved without compromising the graduated compression profile of the stocking itself, which is essential for thrombosis prevention.

As illustrated in Fig. 6C, an 8 × 1 sensing array was constructed, comprising eight horizontally aligned auxetic fibers connected to a custom-engineered wireless data acquisition system. The actual hardware implementation, including the detailed circuit schematic and physical circuit board, and the engineering details for signal acquisition, are comprehensively presented in fig. S30. This integrated system recorded distributed strain signals from the lower limb during multiple activities—sitting (I), standing (II), stooping (III), and exercise (IV)—producing distinct electrical patterns for each state (Fig. 6D). Beyond capturing subtle biomechanical signatures associated with venous status, the integrated array also effectively detected large-scale joint movements, demonstrating its capability to monitor both fine and macroscopic biomechanical activities. To interpret these complex multidimensional datasets, a five-layer artificial neural network (ANN) was developed for intelligent classification (Fig. 6E). Processed multichannel features served as inputs, while successive hidden layers performed nonlinear transformation and feature compression, enabling accurate state prediction in the output layer (*54*). Offline training was used to prioritize recognition accuracy and validate feasibility, laying a foundation for future lightweight edge-computing models. The training curves (Fig. 6F) showed rapid convergence, high accuracy, and minimal loss, confirming the model’s generalization capability without overfitting. Evaluation via a normalized confusion matrix (Fig. 6G) revealed consistently high prediction accuracies exceeding 97% across all categories and reaching 98.5% for stooping and exercise. This performance demonstrates the robust identification of physiological states from complex signal matrices. By accurately decoding strain signatures linked to different motion patterns, this system provides a powerful foundation for venous function assessment and early risk warning. Prolonged static standing, for instance, can elevate venous pressure and promote blood stasis, key risk factors for venous insufficiency. Through recognition of abnormal strain patterns, the ANN model can intelligently identify behavioral risks associated with venous stasis and issue early alerts for potentially harmful activities, such as excessive continuous standing. These results confirm the system’s strong potential for intelligent monitoring of behavioral patterns associated with lower-limb venous health.

## DISCUSSION

In summary, we have developed a bioinspired mechano-adaptive auxetic electronic fiber that orchestrates a dual-modulus helical confinement architecture, yielding programmable mechanical adaptability and a stable negative Poisson’s ratio (ν = −0.47). The resultant fiber demonstrates robust mechanical properties (207.6-MPa strength, 340% elongation) and exceptional durability (>94% electrical retention after 10-day washing cycles). Capitalizing on its auxetic topology, the fiber sensor achieves high microstrain sensitivity (<5%), concurrently generating a substantial electrical output of 8.1 V and an 8.48 mW m^−2^ power density. When configured into distributed body-sensing textiles, it successfully decodes venous motions via embodied intelligence with more than 97% recognition accuracy. This work thereby paves the way for scalable, self-powered, adaptive, and durable biomechanical monitoring systems for advanced next-generation wearable health care.

## MATERIALS AND METHODS

All reagents and materials were of analytical grade and used without further purification. Nature CAs from leather solid waste and AF/dimethyl sulfoxide (DMSO) dispersions were prepared in our laboratory (see notes S1 and S2 for detailed synthesis procedures). The WPU, (3-aminopropyl) triethoxysilane, DMSO, potassium hydroxide, saturated sodium sulfate (Na_2_SO_4_), and multiwalled carbon nanotubes (MWCNTs; diameter: 10 nm, length: 2 μm) were purchased from Macklin Biochemical Co. Ltd. (Shanghai, China). Poly (p-phenylene terephthalamide) fibers were obtained from DuPont. For clarity and reproducibility, the detailed list of all optimized manufacturing parameters, including material formulations, spinning conditions, and structural dimensions, is summarized in table S5.

### Preparation of core fiber

See figs. S4 to S6 and note S3 for the synthesis of the CA/WPU spinning solution. Saturated sodium sulfate (Na_2_SO_4_) aqueous solution was used as the primary coagulation bath. The degassed spinning solution was extruded through a spinneret (orifice diameter: 0.1 to 0.2 mm) into the coagulation bath. Primary drawing (draw ratio: 1.5 to 2.0) was performed within the coagulation bath. Subsequently, the fibers were guided into a secondary water bath (deionized water) to remove residual salt and solvent, ensuring structural stabilization. This was followed by thermal drawing at 80°C (draw ratio: 1.2 to 1.5) within a hot water bath. The resulting fibers were thoroughly washed with deionized water and dried at 40° to 50°C to obtain CA/WPU composite yarns. To introduce electrical conductivity, a functional coagulation bath containing a 5% dispersion of MWCNTs was incorporated into the wet spinning process. The final fibers were dried at 40°C and collected as continuous conductive yarns.

### Preparation of wrapped fiber

The AF/DMSO solution was first subjected to ultrasonic degassing for 30 min to remove entrapped air, followed by wet spinning using deionized water as the primary coagulation bath. The as-spun fibers were collected and dried at 60°C to obtain pure AFs. To introduce electrical conductivity, a second coagulation bath containing a 5% dispersion of MWCNTs was incorporated during the spinning process, functionalizing the AFs with MWCNTs. The resulting conductive fibers were then dried at 60°C and collected. Subsequently, a PDMS coating solution was prepared by mixing the base and curing agent (10:1, w/w) and diluting with hexane (1:10, v/v). The fibers were drawn through this solution at a constant speed of 10 mm/min to ensure a uniform coating, followed by thermal curing at 100°C to obtain the final PDMS-coated wrapped fiber.

### Manufacturing and weaving of auxetic fibers

Auxetic fibers were fabricated using a dc geared turbo motor (model: CHF-GF5560) and a custom-designed winding apparatus. The negative Poisson’s ratio structure was achieved by precisely controlling the feed rate of the core fiber and the rotational speed of the winding mechanism. The auxetic fibers were integrated into commercial compression stockings using a manual weft insertion technique. We adopted a plain weave architecture where the sensor fibers serve as functional weft yarns, interlaced with the warp yarns of the base fabric. To capture the gradient strain distribution, an array of eight fibers was arranged transversely across the calf region, spanning from the ankle to the knee. The length of each sensing fiber was individually tailored to match the graduated limb circumference from the distal to the proximal end. These fibers were positioned at a vertical interval of 5 cm to form a distributed sensing array. For electrical interfacing, the fiber tips were stripped to expose the conductive core and connected to flexible copper wires. The junctions were then encapsulated with flexible textile adhesive. This approach ensures mechanical robustness and stable signal transmission while preserving the intrinsic flexibility of the fabric.

### Characterization and simulation

Surface morphology was characterized using a field-emission scanning electron microscope (FEI Verios 460). Electrical signals were recorded with a Keithley 2635B source measure unit and monitored with a Rigol DS1102E oscilloscope. A portable Moku:Go device (Liquid Instruments) was used for continuous data acquisition and real-time signal processing. Periodic tensile cycling was applied via a commercial linear motor stage. Simulations were performed in COMSOL Multiphysics (v6.1) using the electrostatics module, a geometry based on actual sample dimensions, and a physics-controlled mesh with standard sizing, without introducing user-defined equations. Unless otherwise stated, all mechanical and electrical measurements were replicated on at least three independent samples (*n* = 3) to ensure reproducibility.

## Supplementary Materials

### The PDF file includes:

Figs. S1 to S30 Tables S1 to S5 Supplementary Notes S1 to S7 Legends for movies S1 and S2
