# Supplementary material for: Bioinspired heliconical auxetic biofibers for intelligent biomechanical surveillance
Source: Sci Adv. 2026 Jun 19;12(25):eaed6233. doi: 10.1126/sciadv.aed6233 (PMC13281787; doi:10.1126/sciadv.aed6233)
Supplement: Supplementary file 1 — Figs. S1 to S30 Tables S1 to S5 Supplementary Notes S1 to S7 Legends for movies S1 and S2 [file sciadv.aed6233_sm.pdf]

Science Advances
AAAS

Supplementary Materials for

Bioinspired heliconical auxetic biofibers for intelligent biomechanical surveillance

Yi Zhou *et al.*

Corresponding author: Xuechuan Wang, wangxc@sust.edu.cn; Ouyang Yue, ouyangyue@sust.edu.cn; Xinhua Liu, liuxinhua@sust.edu.cn

Sci. Adv. **12**, eaed6233 (2026)
DOI: 10.1126/sciadv.aed6233

The PDF file includes:

Figs. S1 to S30
Tables S1 to S5
Supplementary Notes S1 to S7
Legends for movies S1 and S2

Other Supplementary Material for this manuscript includes the following:

Movies S1 and S2

Supplementary Figures

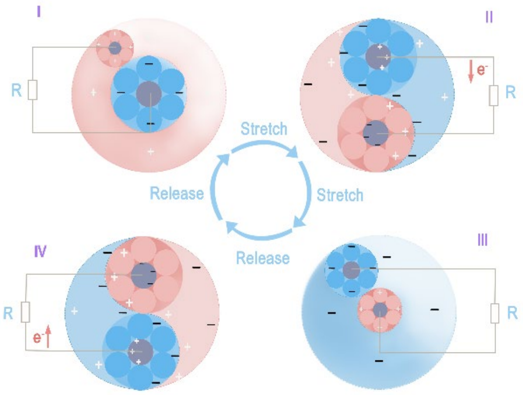

Figure S1. Charge transfer mechanism in the Auxetic-fiber based on a contact-sliding-expansion process. The working principle of the Auxetic-fiber relies on a contact-sliding-expansion mechanism. The core and wrapping yarns act as triboelectric layers, each integrated with a conductive yarn at their center. This design mitigates the charge dilution commonly observed in conventional systems under strain by actively maintaining surface charge density through strain-induced expansion of the contact area, thereby improving charge transfer efficiency. The process involves four stages: Stage I: Initial contact between layers possessing counter charges. Stage II: Under stretching, the wrapping yarn migrates inward, leading to interfacial sliding, growth in contact area, charge separation, and current generation. Stage III: Sliding ceases once the wrapping yarn is fully transitioned, establishing a new charge equilibrium. Stage IV: Elastic recovery restores the yarn to its initial state upon release of the load, repeating the charge rebalancing cycle. The structural self-recovery capability, inherent to the auxetic geometry, ensures consistent triboelectric performance over repeated cycles. Connecting a load between the electrodes results in a potential difference that drives alternating current, yielding continuous AC Output. This approach achieves uniform charge distribution and stable energy conversion through coordinated interfacial dynamics and structural interactivity.

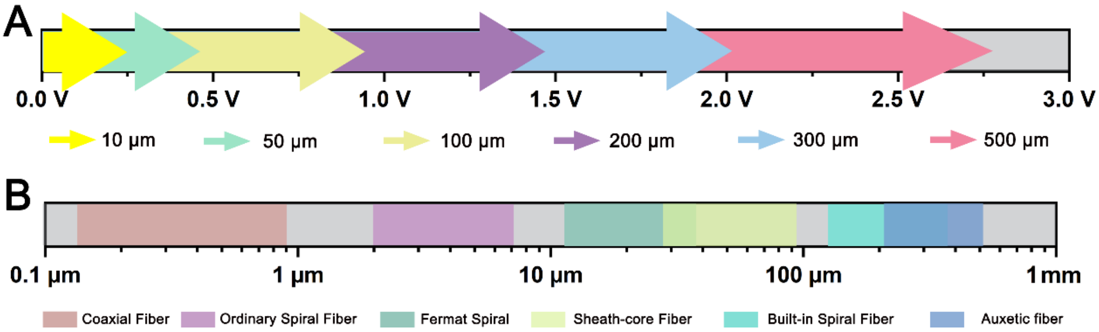

Figure S2. Relationship between the inter-fiber contact distance and Output voltage for different fiber architectures. (**A**) Output voltage of the triboelectric fiber as a function of the contact sliding distance, showing a positive correlation between the Output voltage and the increase in sliding distance. (**B**) Comparative analysis of the achievable contact sliding distance for several triboelectric fiber structures. Owing to

its negative Poisson's ratio effect, the Auxetic-fiber enables a substantially larger friction distance than conventional helical fibers, resulting in a higher Output voltage.

λ(mm)	6	6	5	4.5	4	3.5	3	3
L(cm)	25.6	26	24.6	23.8	21	20	18	18.8

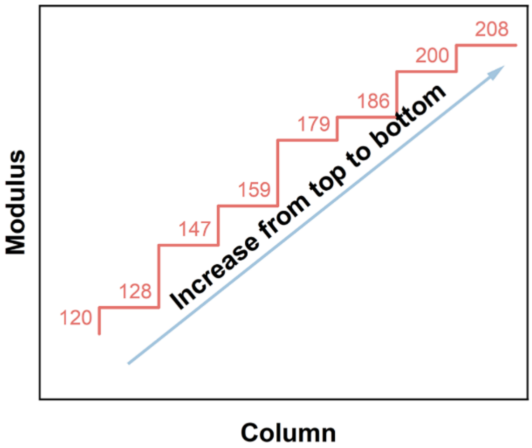

Figure S3. Implementation of a modulus gradient in the Auxetic-fiber (increasing from top to bottom). The associated table details the specific helical pitch (λ) and length combinations (L) employed to create a graded compression profile.

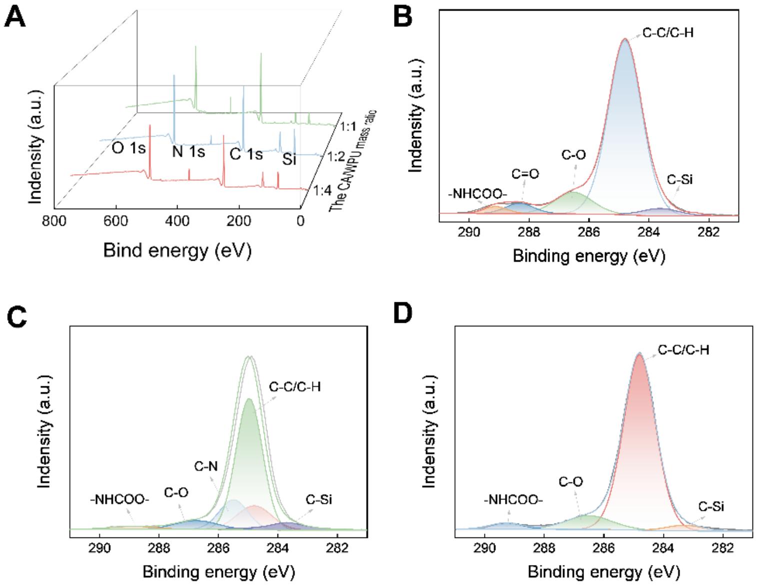

Figure S4. XPS analysis of CA/WPU composite yarns. (**A**) Survey spectra of core yarns with CA-to-WPU molar ratios of 1:4, 1:2, and 1:1. High-resolution C 1s spectra for molar ratios of 1:4 (**B**), 1:2 (**C**), and 1:1 (**D**). The presence of a Si 2p peak confirms the successful incorporation of APTES into the composite. Deconvolution of the C 1s region reveals characteristic bonds at ~284 eV (C–Si) and ~289 eV (N–C=O), verifying the grafting of both APTES and collagen onto the WPU matrix.

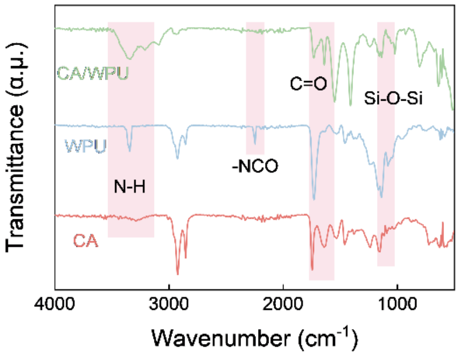

Figure S5. FT-IR analysis of CA/WPU composite yarns. FT-IR spectra of CA/WPU core yarns and their individual components. The N–H stretching vibration at 3340 cm^{-1} broadened and shifted to lower wavenumbers, indicating enhanced hydrogen bonding between WPU –NH groups and collagen C=O or silanol (Si–OH) groups. A notable decrease in the –NCO peak intensity at 2270 cm^{-1} confirmed the consumption of isocyanate groups during reaction. The emergence of a Si–O–Si stretching vibration near 1100 cm^{-1} verified APTES hydrolysis and condensation, yielding a cross-linked structure.

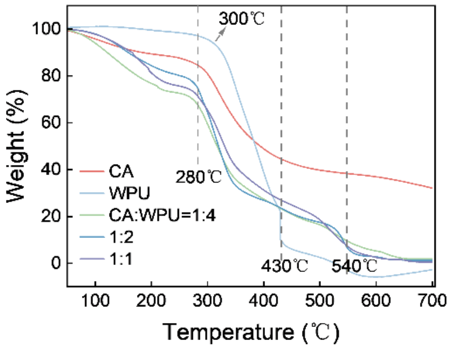

Figure S6. Thermogravimetric analysis (TGA) of CA/WPU core yarns with varying blending ratios. At temperatures below 150 °C, the CA sample showed greater mass loss than pure WPU, attributable to the evaporation of free water. As collagen content increased, the composite yarns exhibited a more gradual mass loss in the 280–430 °C range, suggesting enhanced thermal stability resulting from strengthened interactions between collagen and the WPU matrix.

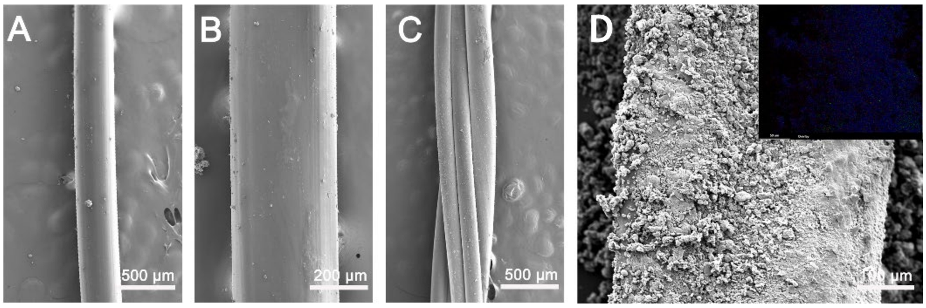

Figure S7. CA/WPU core yarns: (**A, B**) surface morphology of the pristine yarns, (**C**) after twisting, and (**D**) SEM image with corresponding EDS mapping after conductive material deposition.

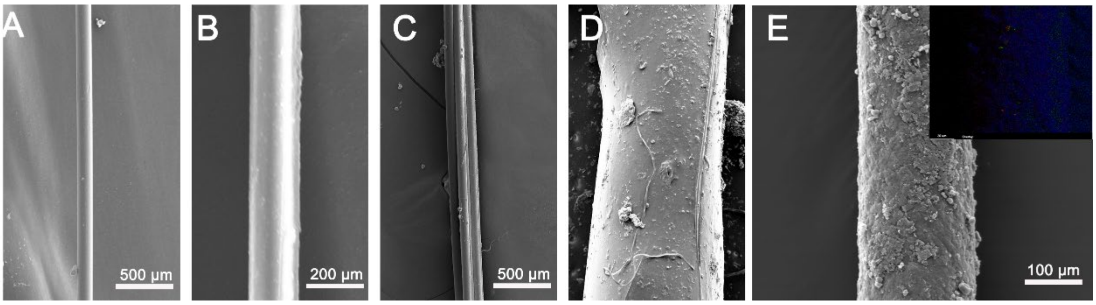

Figure S8. AF/PDMS wrapped yarns: (**A, B**) as-prepared fibers, (**C**) after twisting, (**D**) after PDMS encapsulation, and (**E**) SEM image and EDS mapping following conductive functionalization.

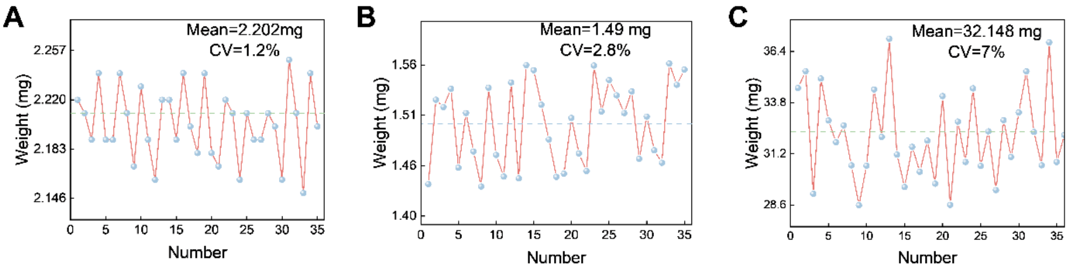

Figure S9. Gravimetric analysis of yarn weight uniformity. (**A**) Weight distribution of CA/WPU core yarns (10 cm length). (**B**) Weight distribution of AF/PDMS sheath yarns. (**C**) Weight distribution of the final Auxetic-fibers. The average weight of 35 randomly selected core yarns was 2.202 mg (CV = 1.2%), and that of the sheath yarn was 1.49 mg (CV = 2.8%). The finished Auxetic-fiber exhibited an average weight of 32.148 mg (CV = 7%). The low coefficients of variation confirm good process controllability and scalability, supporting the reproducible fabrication of textile-based triboelectric sensing networks.

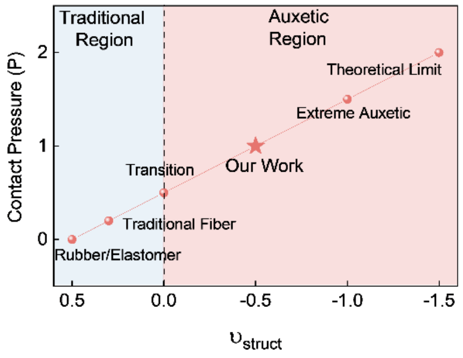

Figure S10. Theoretical correlation between interfacial contact pressure and structural Poisson's ratio. The plot is divided into "Traditional" and "Auxetic" regions. In the traditional region, conventional fibers (e.g., Nylon/Polyester) exhibit minimal Poisson's ratio mismatch, resulting in low contact pressure, while pure soft-sheath structures (e.g., TPU) show zero pressure gain due to synchronous deformation. In the

auxetic region, the Auxetic-fiber (Typical value) achieves a pressure factor 5 times higher than that of ordinary fibers (reference value: 0.2). The curve also delineates the Zero Poisson's ratio state, the Ideal Auxetic structure, and the Theoretical limit, highlighting the pronounced pressure enhancement mechanism enabled by the auxetic design.

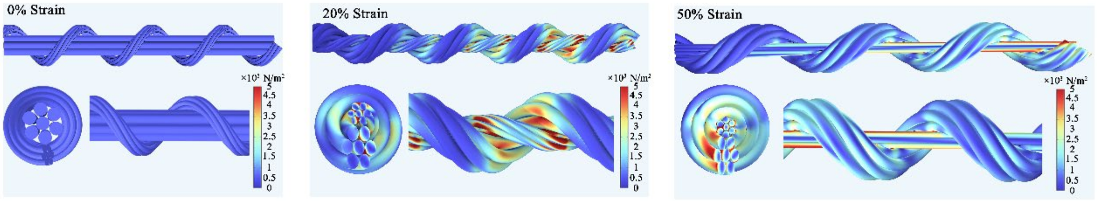

Figure S11. COMSOL simulation of the stress distribution in the low-modulus ratio structure (Control Group). Unlike the high-modulus design, the stress mapping here reveals a uniform and low-intensity stress distribution throughout the helical wrap and core under identical uniaxial radial stretching. The absence of stress concentration at the interface explains the ineffective mechanical-to-electrical conversion efficiency observed in the soft-sheath control group.

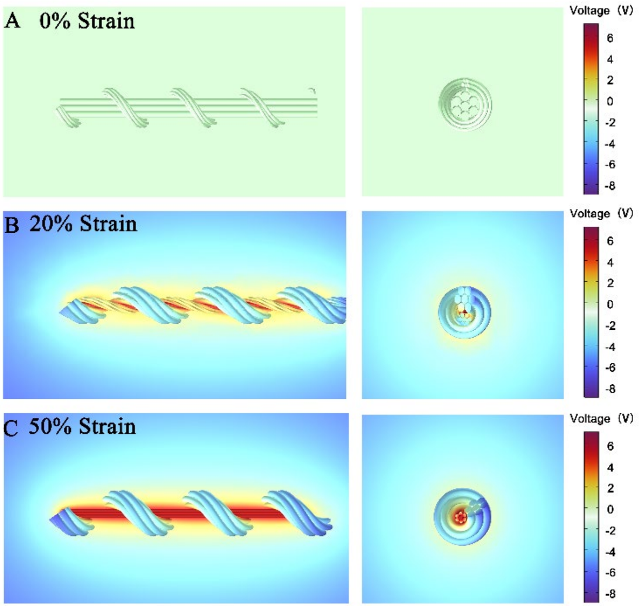

Figure S12. COMSOL simulation of the triboelectric charge transfer mechanism. (**A-C**) The simulation illustrates the potential distribution and surface charge density induced by the contact pressure at the helical interface under 0% (**A**), 20% (**B**), and 50% (**C**) tensile strains. The results indicate that the high contact pressure, resulting from the modulus mismatch, leads to a maximized surface charge density at the contact zones, substantiating the high electrical output observed in the experiments.

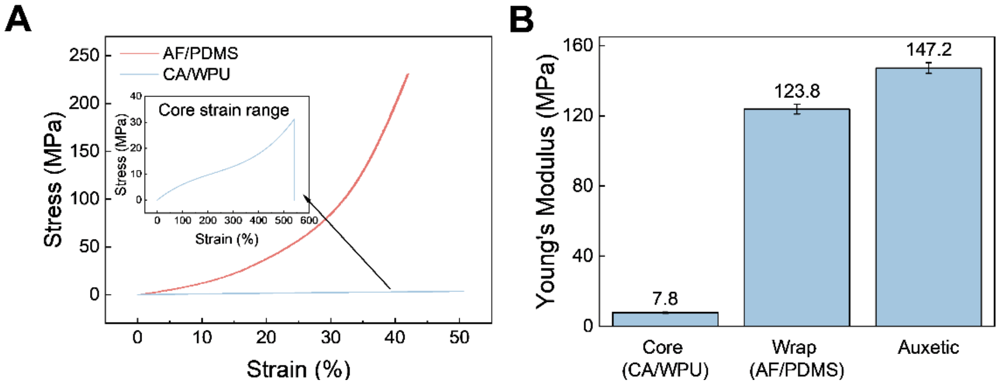

Figure S13. Mechanical characterization and stiffness mismatch analysis of the Auxetic-fiber assembly. (**A**) Stress-strain curves of the core and wrapped fibers up to 50% strain. (**B**) Comparison of Young's modulus for the core fiber, wrapped fiber, and the assembled Auxetic-fiber. The results highlight a critical mechanical mismatch, where the sheath exhibits a considerably higher Young's modulus compared to the soft core. This stiffness disparity serves as the physical origin of the auxetic behavior. During stretching, the stiffer sheath exerts lateral compression on the compliant core. This interaction induces structural reconfiguration and subsequently drives radial expansion. Such expansion effectively optimizes contact area and pressure distribution. Consequently, this mechanism constitutes the fundamental basis for the fiber's enhanced electrical sensitivity and output performance.

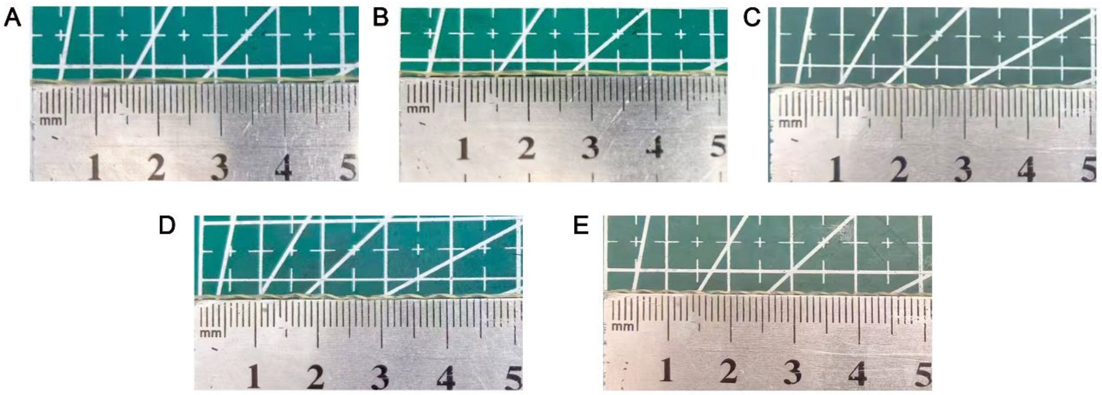

Figure S14. Photographs of the Auxetic-fibers fabricated with different helical pitches (**A**: 10 mm, **B**: 8 mm, **C**: 6 mm, **D**: 4 mm, **E**: 2 mm).

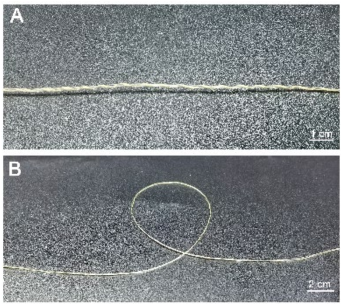

Figure S15. Photograph of the Auxetic-fiber in real form. (**A**) Optical photograph showing the macroscopic morphology of the Auxetic-fiber. (**B**) Photograph of the

Auxetic-fiber under bending deformation. These images demonstrate the fiber's excellent knittability and the structural stability of the bioinspired helical architecture.

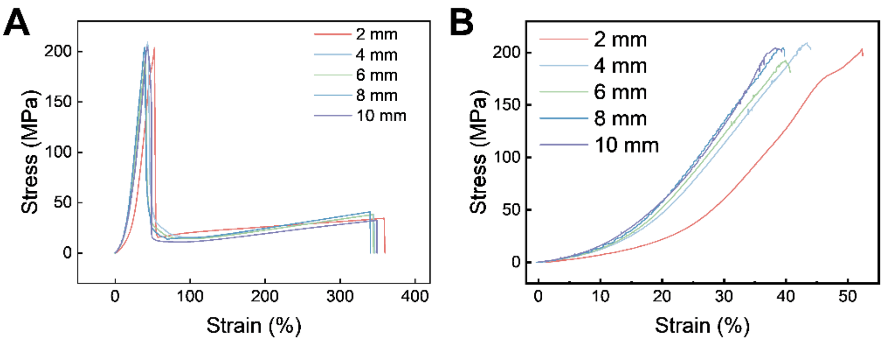

Figure S16. Effect of helical pitch on the tensile performance of Auxetic-fibers. (**A**) Stress-strain curves of Auxetic-fibers produced with different helical pitches. (**B**) Tensile performance of the yarns prior to the first fracture event. As the helical pitch increases, the achievable tensile strain of the Auxetic-fiber decreases, while the tensile strength remains largely unchanged—as the maximum strength is primarily governed by the AF/PDMS wrapping yarn. Conversely, a reduction in helical pitch increases the effective strain distance of the wrapping yarn within the same length, resulting in a corresponding increase in the maximum tensile strain of the Auxetic-fiber.

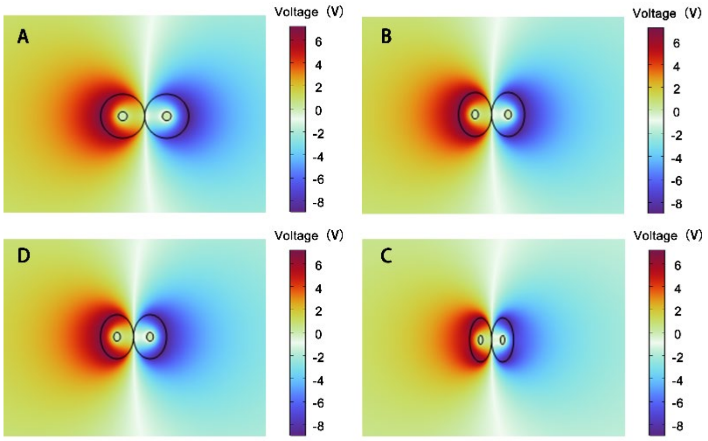

Figure S17. COMSOL simulation (electrostatics module) performed to elucidate the charge transfer mechanism of the Auxetic-fiber under micromechanical deformation. (**A-D**) Simulated electric potential distributions corresponding to four distinct stages of charge generation during a complete stretching cycle: the initial resting state (**A**), the progressive stretching and charge separation stage (**B**), the maximum deformation state (**C**), and the releasing and charge recombination stage (**D**). The simulation reveals that minimal structural alteration occurs in the triboelectric layers during contact. Instead, charge transfer is primarily driven by localized compressive deformation between the layers, which induces spatial variation in the electric potential.

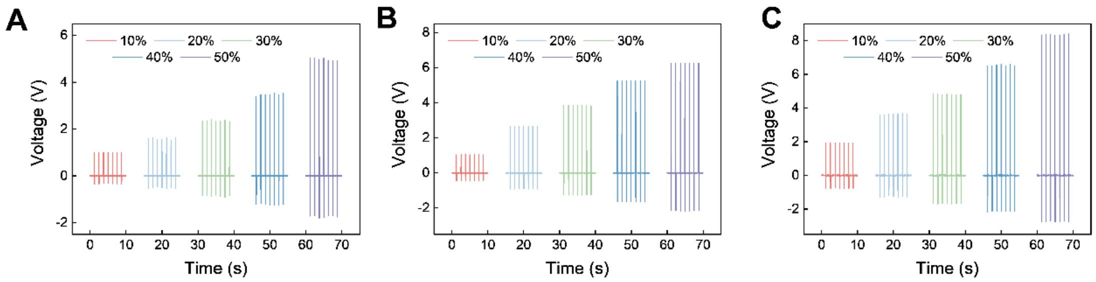

Figure S18. Dependence of the output voltage on the stretching condition for the Auxetic-fibers with different lengths. The fibers exhibit excellent tensile sensitivity across different lengths (**A**: 10 cm, **B**: 15 cm, **C**: 20 cm), with a marked increase in output as length increases, providing a foundation for distributed body-sensing integration.

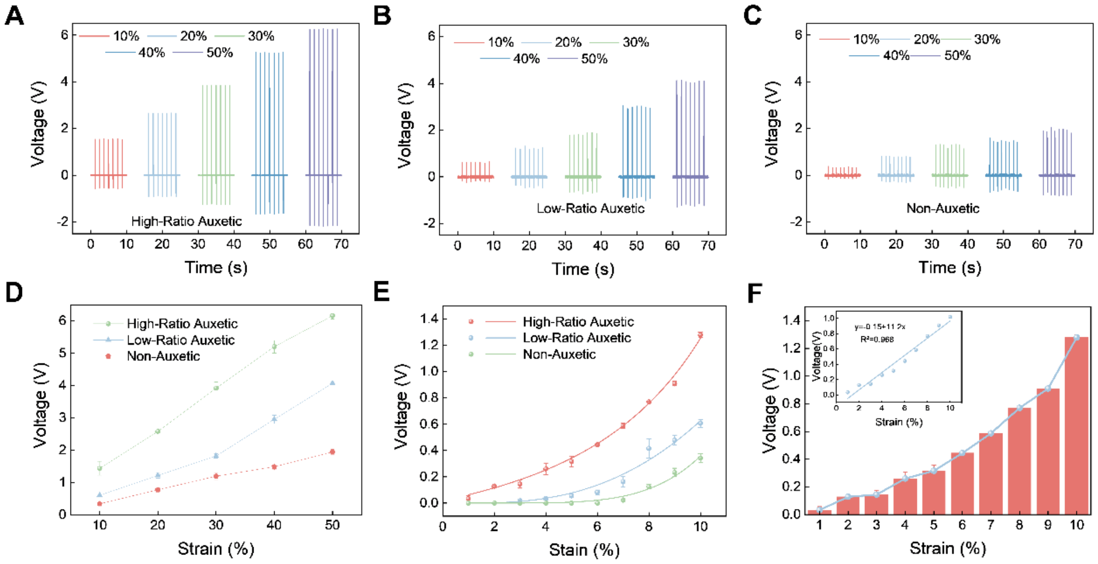

Figure S19. Comparative output performance and sensitivity analysis under dynamic tensile stretching. Real-time open-circuit voltage signals of the (**A**) High-Ratio Auxetic-fiber (Our work), (**B**) Low-Ratio Auxetic fiber (Control), and (**C**) Non-Auxetic twisted fiber (Control) under tensile strains ranging from 10% to 50%. (**D**) Summarized peak voltage output as a function of applied strain (10–50%). The plot illustrates the widening performance gap between the High-Ratio design and the control groups as deformation increases. (**E**) Comparative analysis of output voltage in the low-strain range (0–10%). The solid lines represent curve fitting, highlighting the superior responsiveness of the High-Ratio fiber to micro-deformations compared to counterparts. (**F**) Detailed output performance of the High-Ratio Auxetic fiber under low-strain conditions (< 10%). The bar chart displays the peak voltages at infinitesimal strain increments. The inset shows the linear fitting (R^2=0.968) of the voltage-strain curve, yielding a high strain factor of approximately 11.2, indicating exceptional sensitivity. The High-Ratio Auxetic fiber consistently delivers the highest output across the entire measurement range. At 50% strain, it reaches a peak voltage of approximately 6.16V, which is 1.5 and 3.2 times higher than that of the Low-Ratio and Non-Auxetic counterparts, respectively. This substantial and widening performance gap confirms the

critical role of the optimized high modulus mismatch in maximizing electromechanical conversion efficiency.

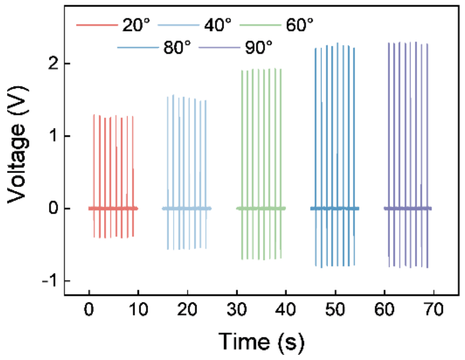

Figure S20. Output voltage of the Auxetic-fiber under different bending angles. The fiber exhibits high sensitivity at low bending angles, while the output signal plateaus at extreme angles (>80°) as the auxetic deformation reaches its spatial limit, restricting further geometric change and thus diminishing the signal variation range.

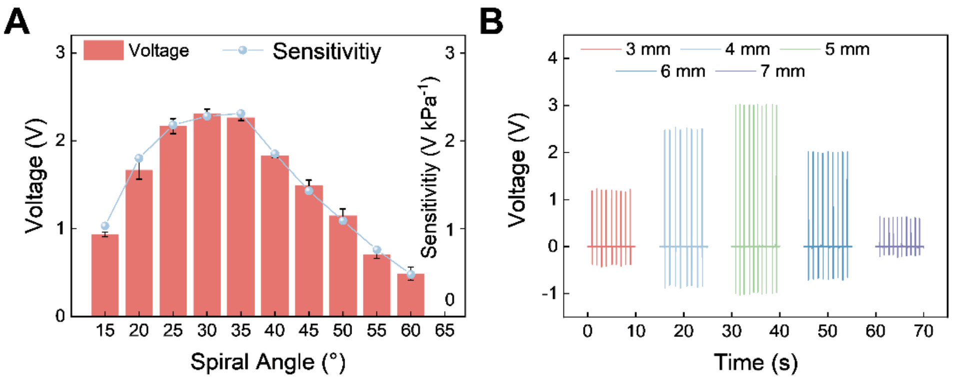

Figure S21. Geometric optimization of helical parameters for maximizing triboelectric sensing performance. (**A**) Output performance and sensitivity of Auxetic-fiber fabricated with different helical angles. (**B**) Output voltage of Auxetic-fibers under different pitch spacings. The length of the fibers is 10 cm. Structural optimization revealed that the triboelectric response depends strongly on the helical geometry: the highest sensitivity (2.31 V·kPa^{-1}) occurred at a helical angle of ~35° (Fig. 4f), while a helical pitch of 5 mm yielded the maximum voltage output (Fig. 4g). Further increases in pitch reduced the signal amplitude, consistent with a diminished Auxetic effect and weaker interfacial coupling.

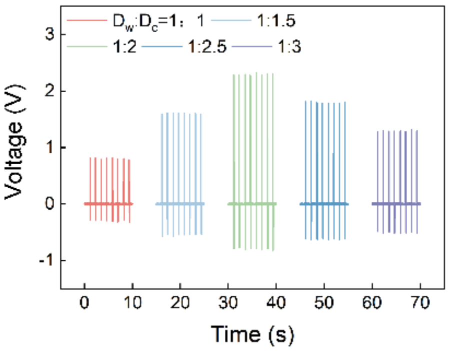

Figure S22. Output voltage as a function of the wrapped-to-core diameter ratio (Dw/Dc = 1:1.5, 1:2, 1:2.5, 1:3). The maximum output voltage was achieved at an optimal diameter ratio of 1:2. This result is attributed to the mechanical interplay where the stiff, thin wrapping sheath most effectively laterally displaces the soft, thick core during elongation, leading to maximal structural expansion and charge separation.

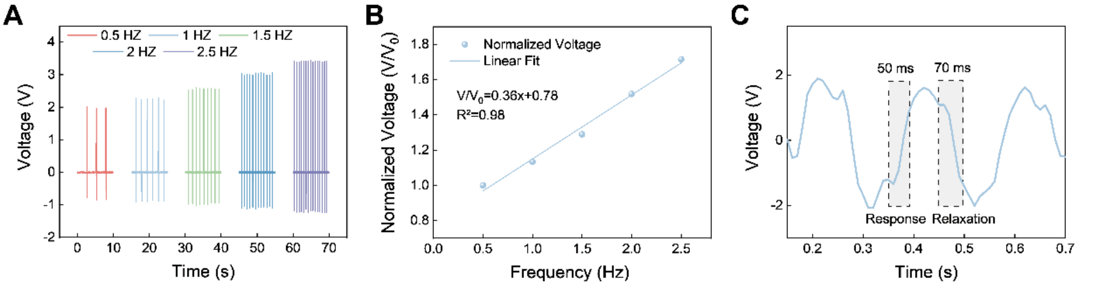

Figure S23. Dynamic response and frequency characterization of the Auxetic-fiber. (**A**) Real-time output voltage signals under varying excitation frequencies ranging from 0.5 Hz to 2.5 Hz. (**B**) Linear fitting analysis of the normalized voltage versus frequency, showing a high linearity (R^2 =0.98). (**C**) Enlarged view of a single signal cycle, identifying a response time of 50 ms and a relaxation time of 70 ms, indicating rapid mechanical-to-electrical conversion suitable for real-time monitoring.

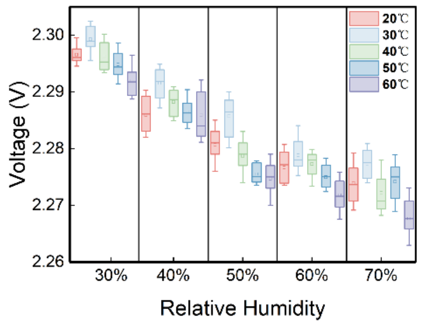

Figure S24. Output voltage of Auxetic-fiber under different temperature and humidity conditions. The output voltage exhibits minimal variation (within 0.2 V), demonstrating its remarkable resilience to ambient conditions.

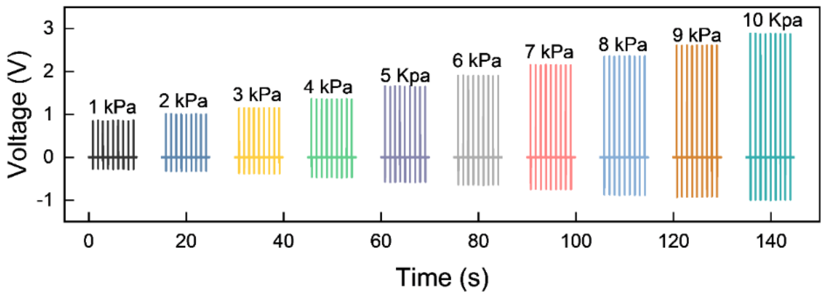

Figure S25. Output voltage of the Auxetic-fiber under different applied pressures. The output voltage exhibits a proportional relationship with applied pressure in the range of 0–10 kPa. A measurable signal variation is maintained at 5 kPa, beyond which the sensitivity increases markedly due to enhanced interfacial contact, demonstrating the fiber's effectiveness across both low and high-pressure regimes.

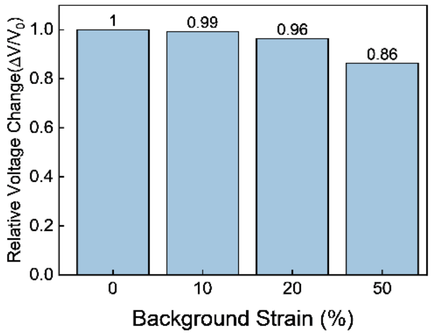

Figure S26. Sensitivity stability under varying background strains. The normalized output amplitude (Δ V/V0) in response to a constant micro-stimulus (1 kPa) was recorded while the Auxetic-fiber was held at fixed tensile strains of 0%, 10%, 20%, and 50%. V0 denotes the response amplitude at 0% strain. The fiber demonstrates high stability, retaining >96% of its sensitivity within the typical physiological working range (0–20%) and maintaining >86% signal retention even under extreme deformation (50%). This confirms that macroscopic tissue stretching does not compromise the detection of microscopic signals.

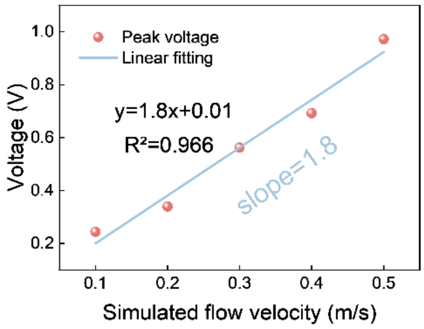

Figure S27. Linear fit between output voltage and simulated blood flow velocity. The plot demonstrates a robust linear correlation ($R^2 > 0.96$) between the fiber's output voltage and fluid velocity within the range of 0.1–0.5 $m \cdot s^{-1}$. This relationship establishes a standard curve for the quantitative calibration of real-time micro-strain signals.

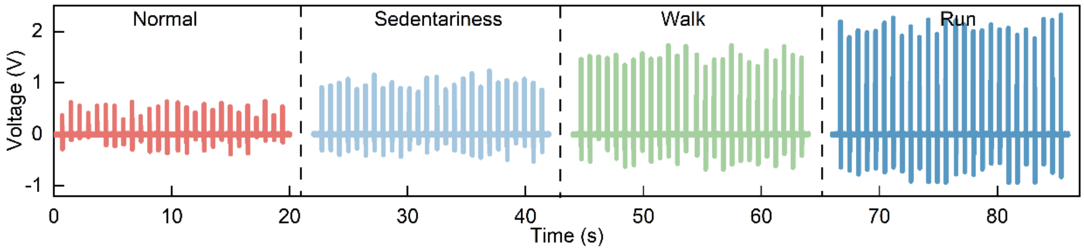

Figure S28. Difference in voltage signals between resting and motion states. Output signals were collected during standing at rest, sedentariness, walking, and running. The signal amplitude increases with movement intensity, yet a discernible response even at rest demonstrates the fiber's high baseline sensitivity for detecting a wide range of physiological states.

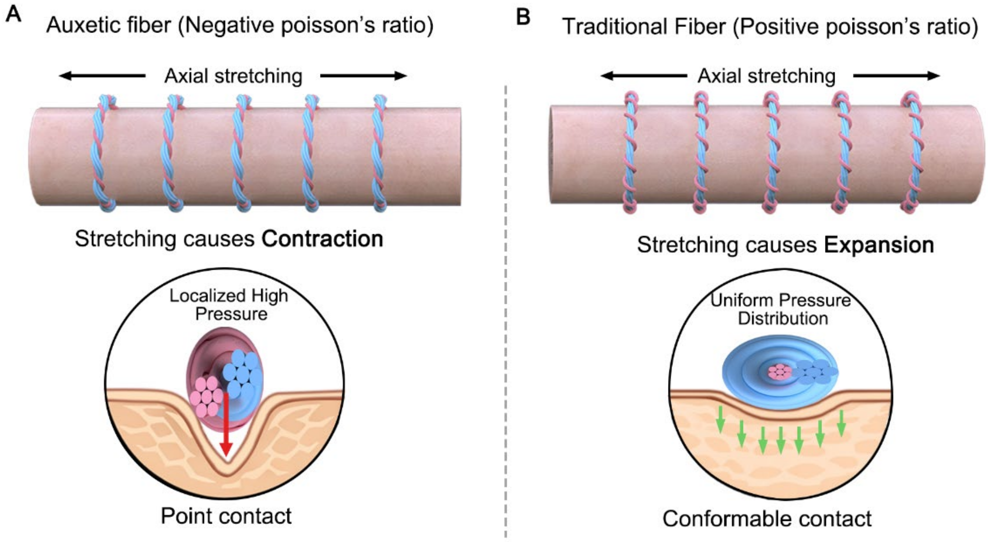

Figure S29. Schematic comparison of the contact mechanics between traditional and Auxetic-fibers on a human limb. (**A**) Traditional fibers with a positive Poisson's ratio exhibit a radial contraction effect under axial stretching. The cross-sectional view reveals a concentrated point-contact mode. This configuration induces high localized pressure that risks restricting blood flow. (**B**) Under axial tension, the Auxetic-fiber undergoes radial expansion. The cross-sectional view illustrates a transition toward a flattened, planar contact interface. This deformation expands the fiber-skin contact area, effectively reducing localized pressure at the interface.

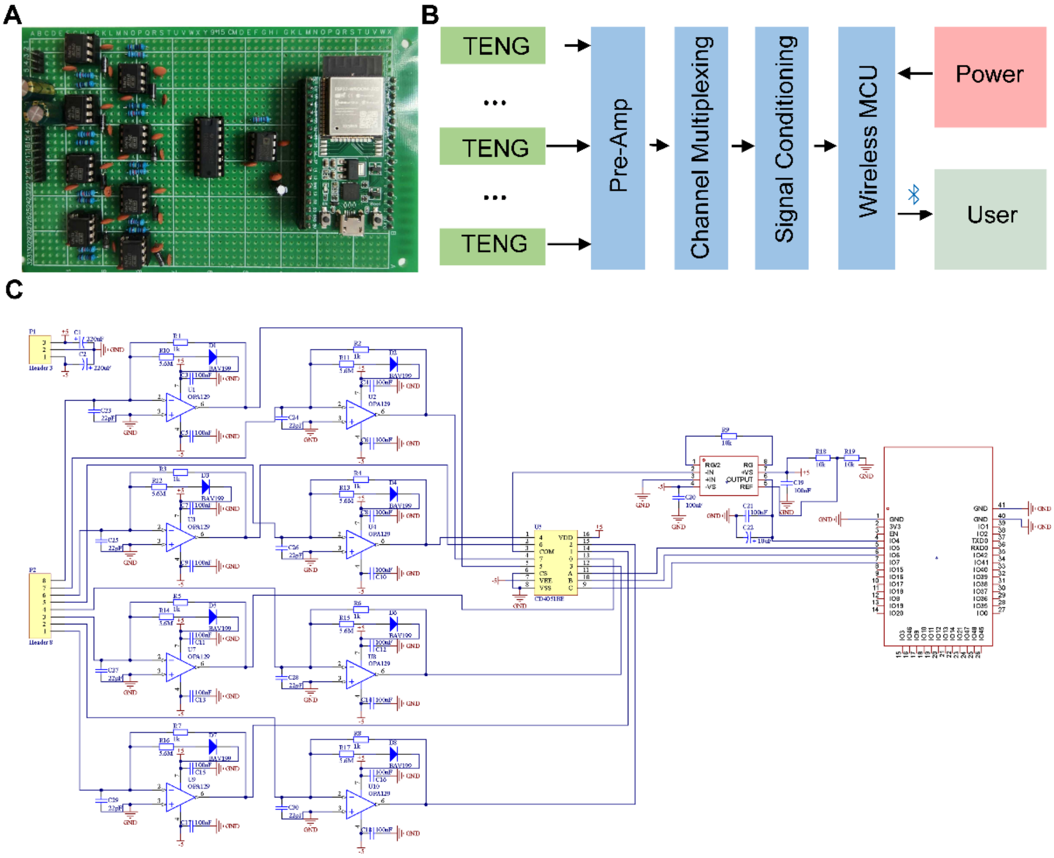

Figure S30. Hardware implementation of the wireless sensing system. (A)

Photograph of the integrated circuit board. (B) Various functional modules integrated into the circuit board. (C) Complete circuit schematic. The architecture features a multi-channel high-impedance analog front-end for charge-to-voltage conversion. It utilizes an analog multiplexer to sequentially route signals (via time-division multiplexing) to a low-noise instrumentation amplifier. Following level-shifting circuitry, the signals are adapted for the single-supply microcontroller and digitized using a built-in 12-bit analog-to-digital converter (ADC). To balance high-fidelity signal capture with power efficiency, the overall system sampling rate is configured at 400 Hz, yielding an effective per-channel acquisition rate of 50 Hz. This ensures the reliable, real-time digitization of low-frequency human biomechanical activities prior to wireless communication.

Table S1: Comparative summary of tensile Output and sensitivity of reported triboelectric fibers.

No.	Refs.	Raw Data	Strain (%)	Max. voltage (V)	Strain Factor
1	Sci Adv, 8(45), eabo0869 (2022).	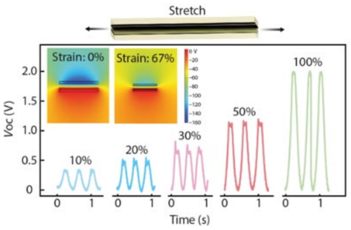	100	2	1.85
2	Adv. Sci. 12, e05363, 2025	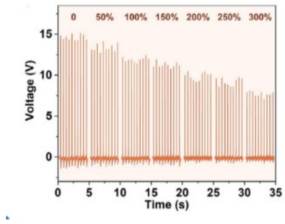	300	15	2.67
3	Adv. Mater., 33(26), 2100782, (2021)	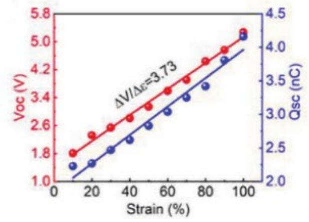	100	5.3	3.73
4	InfoMat, 6(5), e12534, (2024)	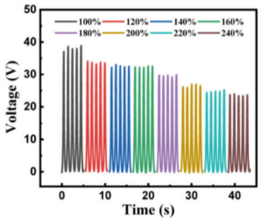	240	39	10
5	Adv. Mater., 35(36), 2300447, (2023).	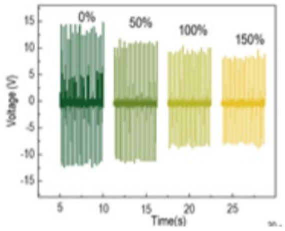	150	7.76	4.13
6	Nano Energy, 136, 110753, (2025)	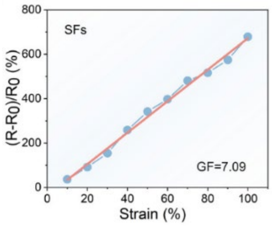	100	–	7.09

7	Adv. Mater., 33(45) 2104681, (2021).	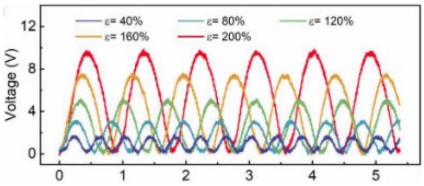	200	9.36	4.9
8	Our work	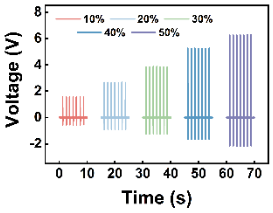	50	6.1	11.75

Table S2: Comparison of fabrication efficiency between our work and state-of-the-art micro-strain sensing fibers.

Ref.	Sensing mechanism	Structure	Fabrication Strategy	Process Complexity	Scalable manufacture	Electrical outputs	Applications
Our work	Triboelectric	Helical Core-wrapped	Standard Wet Spinning and Macroscopic Twisting	Low （Macroscopic preparation and twisting molding）	High (Low-cost and High-speed)	8.1 V (50% strain, 20 cm)	Lower Limb and Venous Health
Nat Commun. 15(1), 2374 (2024).	Triboelectric	Nanofiber buckling structure + Ionogel	Electrospinning + Ionogel injection + Thermal treatment	High (Requires precise control of electrostatic microstructures and shape memory processing)	Medium （Limited by electrospinning efficiency and complex post-processing)	0.9 V (Bending, curvature 6 mm^{-1}. Focus on micro-flexure)	Muscle strength and Pulse/Respiration monitoring
Sci Adv, 8(45), eabo0869 (2022).	Triboelectric	3D Multi-material hollow structure + Liquid metal	Multi-material thermal drawing	Low (One-step drawing, but preform fabrication is time-consuming)	High (Industrial-grade fiber drawing process; kilometer-scale production achievable)	1.5 V (at 67% strain)	Wearable e-textiles and Energy harvesting
Adv. Mater., 33(26), 2100782, (2021)	Triboelectric	Fermat Spiral Nanofibers	Conjugate electrospinning + Mechanical twisting	Medium (Electrospun nanostructures are highly sensitive to environmental conditions and production speed is slow)	Medium (nanostructure fabrication limits mass production speed)	1.8 V (at 50% strain)	Gesture recognition, Waterproof energy yarns
Nano Energy, 136, 110753, (2025)	Triboelectric, Piezoresistive	3D Porous Biomass + Particles	Dip-coating + Encapsulation	Low (Simple dip-coating process based on natural materials)	Medium (Simple process, but consistency of natural materials is difficult to control)	0.3 V (Primarily resistive sensing; TENG output dominates in compression/pressing mode)	Hand motion monitoring, Rehabilitation training
InfoMat, 6(5), e12534, (2024)	Triboelectric	Coaxial Core-Shell	Assembly / Sewing	Medium (Requires core-sheath alignment and interface control.)	High (Materials are accessible, but mass production requires precise process control)	~24 V (Impact under 240% strain. Stretching acts as noise)	Drowning rescue, Vital signs monitoring

Table S3: Comparison of bending monitoring performance of reported triboelectric fibers.

No.	Refs.	Raw Data	Min. Bending Angle	Max. voltage (V)
1	Nat Commun 15(1), 2374 (2024).	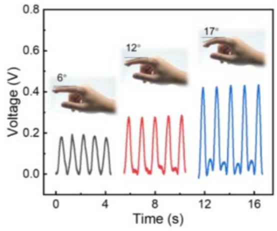	6°-17°	0.4
2	Nat Commun 13(1), 5224 (2022).	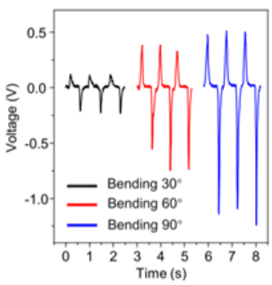	30°-90°	0.5
3	Nat Commun 12(1), 5378 (2021)	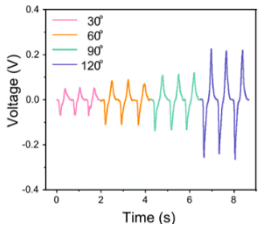	30°-120°	0.22
4	Nat Electron 3(9), 571–578 (2020).	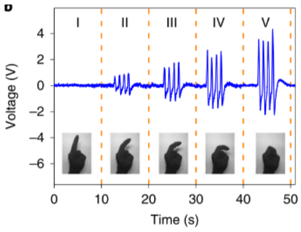	10°-50°	4
5	Our work	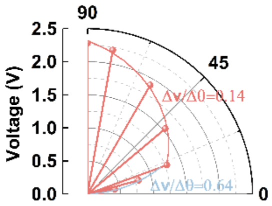	5°-90°	2.28

Table S4: Summary of mechanical parameters and strain factors for fibers with varying modulus ratios.

Group	Component	Young's Modulus (E, MPa)	Mismatch Ratio (E_{wrap}/E_{core})	Strain Factor	Note
1、Optimization Group					
Our Work	Core (CA/WPU)	7.8	≈15.9	11.26	Auxetic
	Sheath (AF/PDMS)	123.8			
Soft-Sheath confinement fibers	Core (CA/WPU)	7.8	≈2	6.7	control group(materials)
	Sheath (Soft TPU)	17.2			
Traditional Twisted fibers	Core (CA/WPU)	7.8	≈15.9	3.28	control group(structure)
	Sheath (AF/PDMS)	123.8			
2. Ratio Discussion Group					
Ratio B (Medium)	Core (CA/WPU)	7.8	≈5	8.21	control group
	Sheath (Spandex)	40.2			
Ratio C (High)	Core (CA/WPU)	7.8	≈20	10.52	control group
	Sheath (TPU)	160.3			
Ratio D (High)	Core (CA/WPU)	7.8	≈50	10.81	control group
	Sheath (PTFE wire)	390			
Ratio D (High)	Core (CA/WPU)	7.8	≈100	11.09	Low elongation
	Sheath (HDPE)	800			

Table S5: Summary of the optimized materials, fabrication parameters, and geometric dimensions for the Auxetic-fiber.

Component	Materials	Key Parameter	Optimized Value
Core Fibe	CA / WPU	Blending Ratio (wt/wt)	1 : 4
		Coagulating bath	Saturated Na_2SO_4 aqueous solution
		Needle Gauge	20 G
		Extrusion Pressure	Pneumatic control (~ 1.2 bar)
		Drying	40°C
wrapped fiber	AF / PDMS	AF Concentration	10%
		Needle Gauge	25 G
		Extrusion Pressure	Pneumatic control (~ 0.8 bar)
		Coagulating bath	Deionized Water
		Withdrawal Speed	30 mm/min
		Curing temperature	60°C
	PDMS Coating	PDMS Formulation	Base : Agent=10 : 1
		Solvent Dilution	1 : 10
		Drawing Speed	10 mm/min
		Curing temperature	100°C
Auxetic Structure	Helical	Helical Pitch	5.0 ± 0.66 mm
		Helical Angle	35 ± 4°
		Modulus Ratio (E_w/E_c)	≈15
		wrapped-to-core diameter ratio	1 : 2
Textile Integration	commercial anti-embolism stocking	Integration Method	Manual Weft Insertion and Plain Weave Pattern
		Array Layout	1×8 Transverse Array
		Fiber Spacing	4.0 cm
		Fiber Length	Customized to local limb circumference
		Encapsulation	Flexible Fabric Glue

Supplementary Note 1: Preparation of Collagen Aggregates.

Leather solid waste was cut into uniformly sized pieces and immersed in 1 M acetic acid overnight until fully swollen and translucent. The swollen tissue was thoroughly rinsed with deionized water to remove residual acetic acid. The treated tendon fragments were then mixed with 0.1 M acetic acid in a beaker to form a pre-dispersion of collagen aggregates. This pre-solution was homogenized using a commercial high-speed blender (JX-08) at 20,000 rpm for 5 minutes per cycle, repeated every 10 minutes over a total duration of 1 hour. The resulting collagen aggregates were dissolved in 0.1 M acetic acid under continuous stirring at 1000 rpm for 1 hour. The solution was centrifuged at 8000 rpm to remove insoluble components. The supernatant was collected and lyophilized to obtain collagen powder.

Supplementary Note 2: Preparation of ANF/DMSO solution.

Para-aramid fibers (10 wt%) were subjected to mechanical refining for 1 hour using a laboratory refiner to produce aramid nanofibers (ANFs). The resulting fibrillated fibers were then dried in a vacuum oven. Subsequently, the PPTA fibers and potassium hydroxide were dispersed in dimethyl sulfoxide (DMSO). The mixture was magnetically stirred at room temperature for 7 days, yielding a homogeneous, transparent dark red ANF/DMSO solution. A reaction period of 7 days provided the optimal solution for spinning.

Supplementary Note 3: Preparation of CA/WPU Composite Spinning Solution.

Collagen powder was dissolved in 0.5 M acetic acid solution under low-speed magnetic stirring (300 rpm) for 2–3 hours until complete dissolution to obtain a homogeneous collagen/acetic acid solution. APTES was mixed with deionized water at a 1:9 (v/v) ratio, and the pH was adjusted to 4.5–5.0 using a few drops of dilute acetic acid. The mixture was stirred magnetically at room temperature for 30 minutes to allow full hydrolysis of APTES into reactive silanol groups. An aqueous polyurethane elastic emulsion (60% solid content) was diluted with deionized water to a solid content of 20–25% to reduce viscosity for improved spinnability. The diluted WPU solution was slowly adjusted to pH 8.5–9.0 using 0.1 M ammonium hydroxide under gentle stirring. The hydrolyzed APTES solution was added dropwise at 1 mL/min into the pretreated WPU solution maintained at 40°C with constant stirring (500 rpm). emulsion (60% solid content) was diluted with deionized water to a solid content of 20–25% to reduce viscosity for improved spinnability. The diluted WPU solution was slowly adjusted to pH 8.5–9.0 using 0.1 M ammonium hydroxide under gentle stirring. The hydrolyzed APTES solution was added dropwise at 1 mL/min into the pretreated WPU solution maintained at 40°C with constant stirring (500 rpm). After complete addition, the reaction was continued for 4–6 hours at 40°C. The resulting WPU-APTES prepolymer solution was cooled below 10°C in an ice-water bath. The collagen/acetic acid solution was then added dropwise at 0.5 mL/min into the cooled WPU-APTES prepolymer under vigorous stirring (800–1000 rpm). Simultaneously, PBS buffer was slowly introduced via a separate feed pump to adjust the final pH to 7.0–7.5. The final CA/WPU spinning solution was subjected to ultrasonic degassing for 30 minutes to remove air bubbles.

Supplementary Note 4: Geometric Relationship in the Helical Wrapping Process.

The core fiber advanced axially through bottom traction, while the wrapped fiber—mounted on a rotating bobbin—was helically wound at a controlled angle determined by the ratio between feeding speed and rotational velocity, as defined by the following equation:

$$tan\theta = \frac{\omega\varphi}{2\upsilon} \quad (1)$$

$$\lambda = \frac{2\pi\upsilon}{\omega} \quad (2)$$

Where, θ is the winding angle between the wrapped fiber and the horizontal axis of the core fiber, ω is the angular velocity of the wrapped fiber, φ is the diameter of the resulting Auxetic-fiber, υ is the feed rate of the core fiber, and λ is the helical pitch of Auxetic-fiber.

Supplementary Note 5: Derivation of the contact area variation between the wrapping and core yarns during the stretching of the Auxetic-fiber.

Stage 1: Initial Stretching

In the initial stage, the helical wrapping yarn gradually transforms into a double-helix structure. The contact area is governed by the geometric relationship between the wrapping and core yarns. The contact chord length in the cross-section is given by:

$$L = 2\sqrt{{r_w}^2 - (d_0 - \beta\epsilon - r_c)^2} \quad (3)$$

The length of the helix in one pitch is:

$$s = \lambda(1+\varepsilon)\sqrt{1 + \left(\frac{k_h}{1+\varepsilon}\right)^2} \quad (4)$$

where the helical tightness parameter $k_h = \frac{2\pi r_w}{\lambda}$ is related to the initial helical angle.

The contact area per pitch is proportional to the chord length multiplied by the helical length (assuming uniform contact width):

$$A_{P1} = L \times S = 2\sqrt{r_w^2 - (d_0 - \beta\epsilon - r_c)^2} \times \lambda\sqrt{(1+\varepsilon)^2 + k_h^2} \quad (5)$$

Considering n wrapping yarns, the total contact area becomes:

$$A_1 = 2n\sqrt{r_w^2 - (d_0 - \beta\varepsilon - r_c)^2}\frac{\lambda(1+\varepsilon)}{\cos\theta} \quad (6)$$

Stage 2: Double-Helix Formation

In the second stage, a double-helix forms between the wrapping and core yarns, accompanied by positional exchange. The contact chord length is based on the radii of the two yarns and the center distance:

$$L = 2\sqrt{r_w + r_c + \delta^2 - (d_0 - \beta\epsilon)^2} \quad (7)$$

The contact area includes the contact width. Ideally, the contact width is proportional to the chord length, so the area is proportional to the square of the chord length. The contact area per pitch is:

$$A_{P2} = k_w \times L^2 \times S = k_w\left[2\sqrt{r_w + r_c + \delta^2 - (d_0 - \beta\epsilon)^2}\right]^2 \times \lambda\sqrt{(1+\varepsilon)^2 + k_h^2} \quad (8)$$

With n wrapping yarns, the total contact area is:

$$A_2 = nk_w\left[2\sqrt{r_w + r_c + \delta^2 - (d_0 - \beta\varepsilon)^2}\right]^2 \frac{\lambda(1+\varepsilon)}{\cos\theta} \tag{9}$$

Parameter Definitions:

n : number of wrapping yarns

r_w : radius of the wrapping yarn

r_c : radius of the core yarn

d_0 : initial center-to-center distance between wrapping and core yarns

β : reduction rate of center distance during strain

ε : axial strain

λ : initial helical pitch

θ : helical angle

k_w : dimensionless scaling factor for contact width in Stage 2

δ : initial contact deformation

k_h : helical tightness parameter ($k_h = \frac{2\pi r_w}{\lambda}$).

Supplementary Note 6: Derivation of the effective contact distance between the wrapping and core yarns during the stretching of the Auxetic-fiber.

The theoretical maximum contact distance of the Auxetic-fiber, defined as the distance at complete separation, is given by:

$$d_{max} = d_0 - r_c - r_w \tag{10}$$

Due to the surface roughness of the Auxetic-fiber, the actual contact state is governed by the contact ratio:

$$\gamma = 1 - \frac{A}{2\pi r_w L(1+\varepsilon)} \tag{11}$$

By combining the helical geometry with the tensile behavior of the Auxetic-fiber, the inter-yarn contact distance can be derived as:

$$d = \left(1 - \frac{A}{2\pi r_w L(1+\varepsilon)}\right)(d_0 - \beta\varepsilon) - (r_c + v d_0 - r_w) \tag{12}$$

Where, L is the effective length of the yarn, v is the Poisson's ratio of the yarn structure, r_w and r_c the radii of the wrapping and core yarns, respectively, γ is the contact ratio. d_0 is the initial center-to-center distance between the yarns, β is the reduction in the center distance during straining, and ε is the applied axial strain.

Supplementary Note 7: Theoretical Derivation of Interfacial Pressure between Core and Wrapping Fibers under Tension.

To quantitatively elucidate the mechanism by which the auxetic structure enhances the triboelectric output, we derived the analytical relationship between the interfacial contact pressure (P) and the structural parameters, specifically the Poisson's ratio and elastic modulus.

Stage 1: Geometric Model and Fundamental Assumptions.

We model the Auxetic-fiber as a simplified concentric cylindrical system consisting of a soft core layer (radius R, Young's modulus E_c, Poisson's ratio ν_{core} and a stiff wrapping layer (equivalent Young's modulus E_w, equivalent structural Poisson's ratio ν_{struct}).

Assumption 1: The deformation occurs within the linear elastic range.
Assumption 2: The stiffness of the wrapping layer is markedly greater than that of the core ($E_W \gg E_c$), acting as a semi-rigid constraint boundary.
Assumption 3: The fiber is subjected to a uniform axial tensile strain ε_z.
Stage 2: Radial Displacement Mismatch: The Origin of Pressure.
Upon applying an axial tensile strain ε_Z, both the core and sheath layers exhibit a tendency for radial deformation. According to the definition of Poisson's ratio $(\nu = -\varepsilon_r - \varepsilon_z)$.
Natural radial displacement of the core (unconstrained), since the core is a soft material, it tends to contract severely:

$$\mathcal{U}_{r,c}^{free} = -Rv_{core}\varepsilon_z \tag{13}$$

Natural radial displacement of the sheath (unconstrained):

$$\mathcal{U}_{r,w}^{free} = -R\nu_{\mathrm{struct}}\varepsilon_z \tag{14}$$

Radial Interference (δ):
The discrepancy in free deformation trends between the core and sheath creates a potential geometric mismatch. For our helical interlocking structure, this mismatch forces an interaction between the layers. We define this "radial interference" as the difference in their natural displacements:

$$\delta = \mathcal{U}_{r,w}^{free} - \mathcal{U}_{r,c}^{free} \tag{15}$$

$$\delta = R\varepsilon_z\left(\nu_c - \nu_{\nu_{\mathrm{struct}}}\right) \tag{16}$$

Stage 3. Derivation of Contact Pressure Based on Lamé's Solution.
According to the deformation compatibility condition, the core and sheath must remain in contact at the interface. Consequently, an interfacial contact pressure P is generated, which compresses the core and pushes against the sheath to eliminate the geometric mismatch δ.Based on the theory of thick-walled cylinders (Lamé's solution) and the interference fit model, the radial elastic deformation $(\mathcal{U}_p)$ caused by the internal/external pressure P can be approximated as:

$$\delta = \mathcal{U}_{p,\ c} + \mathcal{U}_{p,w} \tag{17}$$

$$\delta = \frac{P\,R}{E_c} + \frac{P\,R}{E_w} \tag{18}$$

Solving for the contact pressure P:

$$P = \frac{\varepsilon_Z(\nu_{core} - \nu_{\mathrm{struct}})}{\frac{1}{E_c} + \frac{1}{E_w}} \tag{19}$$

Stage4. Simplification via Modulus Mismatch Effect.
Since we utilized a high-modulus Aramid/PDMS sheath to wrap a low-modulus Collagen/WPU core, the condition $E_W \gg E_c$ is satisfied. Therefore, the term $\frac{1}{E_W}$ in the

denominator is negligible compared to $\frac{1}{E_c}$. The formula simplifies to:

$$P \approx E_{core}\varepsilon_Z(\nu_{core} - \nu_{\text{struct}}) \quad (20)$$

Given that the triboelectric output voltage V_{out} is positively correlated with the contact pressurePin the low-pressure regime, we obtain the final qualitative proportional relationship:

$$V_{out} \propto E_c\varepsilon_Z\left(\nu_{core} - \nu_{\nu_{\text{struct}}}\right) \quad (21)$$

Stage 5. Physical Interpretation

This derivation identifies two necessary conditions for achieving high-sensitivity micro-strain sensing:

1. Maximization of Poisson's Ratio Difference $(\nu_{core} - \nu_{\text{struct}})$:

For conventional fibers, $\nu_{\text{struct}} > 0$ (positive Poisson's ratio). The difference with ν_{core} is minimal, resulting in weak or negligible contact pressure (potentially leading to gap formation). In contrast, for the Auxetic-fiber in this work, $\nu_{\text{struct}} < \nu_c$. This dramatically amplifies the difference term, theoretically boosting the contact driving force by several fold.

2. Rigid Constraint Mechanism $E_w \gg E_c$:

The formula indicates that the generation of pressure P contingent upon the denominator $\frac{1}{E_w}$ being sufficiently small. If the wrapping layer were too soft, the denominator would increase, diminishing the pressure P. This theoretically validates the necessity of the "high-modulus warp/low-modulus core" dual-modulus helical constrained wrapping design for maintaining high-pressure contact.

Supplementary Movie Legends:

Movie 1: Real-time output signal of the Auxetic-fiber during stretching.

This video illustrates the scalable electrical performance of the Auxetic-fiber. The system continuously records and displays the real-time open circuit voltage as the Auxetic-fiber undergoes tensile deformation. The dynamic electrical output increases proportionally with the fiber length. A 15 cm sample reaches a peak voltage of approximately 2.5 V at a 20% strain. This visual evidence confirms the capability of the structural design with a high modulus ratio to stably and continuously generate triboelectric charges under macroscopic deformations.

Movie 2: Bioinspired fluid pressure testing system for characterizing the Auxetic-fiber under micro-strain.

The video demonstrates the operation of the bioinspired fluid pressure testing platform. The Auxetic-fiber is tightly wrapped around a compliant silicone tube. Localized fluid pressure induces dynamic radial expansion of the tube wall as fluid is pumped through the tube at controlled velocities. This expansion represents a volumetric micro-strain. The rapid signals displayed on the monitor represent the real-time triboelectric voltage outputs generated by the fiber in direct response to these flow-induced mechanical expansions. The amplitude of these synchronized voltage peaks increases monotonically with fluid velocity. This relationship illustrates the high sensitivity of the sensor in capturing subtle dynamic mechanical signatures.
